# Axl inhibitor-mediated reprogramming of the myeloid compartment of the *in vitro* tumor microenvironment is influenced by prior targeted therapy treatment

**DOI:** 10.3389/fimmu.2025.1601420

**Published:** 2025-06-05

**Authors:** Anisha Datta, Laura C. Bahlmann, Diana N. Gong, Erin N. Tevonian, James B. Lorens, Douglas A. Lauffenburger

**Affiliations:** ^1^ Department of Biological Engineering, Massachusetts Institute of Technology, Cambridge, MA, United States; ^2^ Department of Biomedicine and Centre for Cancer Biomarkers, University of Bergen, Bergen, Norway

**Keywords:** Axl, macrophage, dendritic cell, tumor microenvironment, immunotherapy, targeted therapy, melanoma

## Abstract

Axl, a member of the receptor tyrosine kinase family comprised of Tyro3, Axl, and MerTK, is a promising cancer therapeutic target actively under clinical investigation. Axl is understood to be a dual target in cancer to (1) prevent tumor cell growth and invasion and (2) potentiate anti-tumor immunity. This immunity is characterized by myeloid cell activation and downstream recruitment and activation of anti-tumor T cells. However, the ways by which Axl inhibition promotes myeloid cell activation in the tumor microenvironment are incompletely understood. There is thus a need to understand the effects of Axl inhibition on myeloid cells in the context of the broader tumor microenvironment. Here, we developed a human *in vitro* model system using primary human monocyte-derived macrophages, primary human monocyte-derived dendritic cells, and Axl-expressing melanoma tumor cells to elucidate the effects of Axl inhibition on the myeloid compartment of the tumor microenvironment. We found that treatment with the Axl-specific small molecule inhibitor bemcentinib yields increased expression of markers of activation in both macrophages and dendritic cells. Interestingly, the addition of dendritic cells to the system appears to dampen macrophage response, suggesting that these cells cooperate to share the burden of the innate immune response. Most importantly, we found that treatment-naïve tumor cells and targeted therapy-treated tumor cells have distinct impacts on macrophage state, and these differences dictate the nature of the immune cell response to Axl inhibition. As a whole, our work highlights the utility of *in vitro* models in unraveling the complex mechanistic effects of Axl inhibition and establishes a robust model system that can be used in future mechanistic drug studies with the potential to inform clinical trial design.

## 1 Introduction

Immune checkpoint blockade therapy has revolutionized cancer therapy, demonstrating the power of galvanizing a patient’s own immune system against their tumor (1). However, like other modalities of cancer therapeutics, the efficacy of this form of immunotherapy is limited to a subset of patients. Patients responsive to immune checkpoint blockade have immunologically “hot” tumors characterized by T cell infiltration prior to administration of this immunotherapy (2). In contrast, immunologically “cold” tumors, which lack this prerequisite T cell infiltration, do not respond. There is thus a need to develop therapies that transform immunologically “cold” tumors to “hot” ones to extend the span of patients who will benefit from and respond to immune checkpoint blockade.

**Figure 1:**
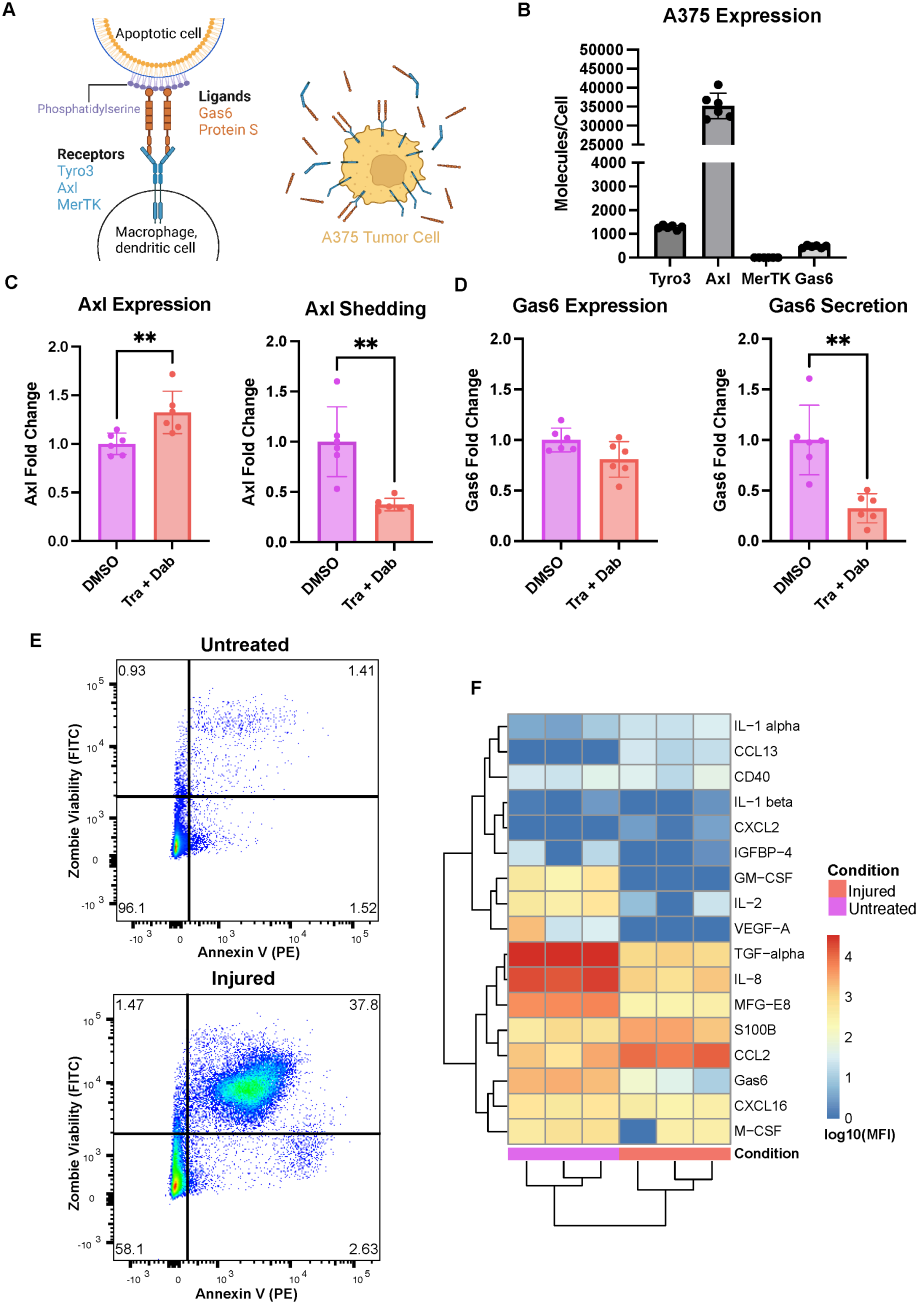
The A375 malignant melanoma cell line expresses Axl and responds to trametinib + dabrafenib treatment. **(A)** Schematic of receptor-ligand dynamics of Axl/TAMRs with respect to efferocytosis (left). A375 tumor cells have cellular Axl and Gas6 that can be measured in cell lysates in addition to shed Axl and secreted Gas6 that can be measured in media supernatants (right). Created with BioRender.com. **(B)** A375 cells express high Axl as compared to Tyro3 and Gas6, and express no MerTK measured via ELISA on cell lysates. n = 3 each in 2 independent experiments. **(C)** Treatment with trametinib + dabrafenib (Tra + Dab) yields increased Axl expression and decreased Axl shedding measured via ELISA on cell lysate (left) and supernatant (right), respectively. n = 3 each in 2 independent experiments. **p < 0.01 Mann-Whitney U test. **(D)** While trametinib + dabrafenib treatment does not change A375 Gas6 expression as assayed via ELISA on cell lysate (left), this treatment does decrease Gas6 secretion (right). n = 3 each in 2 independent experiments. **p < 0.01 Mann-Whitney U test. **(E)** Representative flow cytometry dot plots of untreated and injured A375s’ viability. Annexin V binding on the x-axis is indicative of phosphatidylserine exposure while Zombie Green Viability Dye on the y-axis is indicative of a compromised cell membrane. **(F)** Cytokines, chemokines, and growth factors detected in the media of untreated and injured A375s are distinct, measured via Luminex. n = 3 independent experiments.

Axl, a receptor tyrosine kinase belonging to the family comprised of the Tyro3, Axl, and MerTK receptors (TAMRs), is an attractive target for this purpose (3, 4). Axl is widely recognized and has been extensively studied as a target in cancer therapy due to its role in tumor cell proliferation, survival, migration, and invasion (5–12). It has also been implicated in therapy resistance (13–18) and immune evasion (19–22). As a result, Axl is a target in many ongoing clinical trials in a variety of cancer indications (23, 24). However, Axl expression and signaling is not exclusive to the diseased tumor cells. Physiologically, the TAMRs and their ligand Gas6 are crucial to the innate immune system (3, 25–27). Macrophages and dendritic cells, the myeloid cells tasked with coordinating T cell recruitment and activation, are phagocytes that constantly clear cellular debris (28). Their expression of the TAMRs and Gas6 enable them to conduct efferocytosis, which is the non-immunogenic clearance of apoptotic cells. Specifically, Gas6 acts as a bridging molecule between phosphatidylserine on an apoptotic cell and TAMRs on the phagocyte (**Figure 1A** left), and the receptor clustering that results from this leads to downstream signaling in the phagocyte to (1) consume and degrade the apoptotic cellular debris and (2) prevent inflammation (29). Therefore, Axl expression and signaling in these innate immune cells dampens the immune response, meaning its inhibition in the tumor microenvironment may not only lead to tumor cell death but also result in macrophage and dendritic cell activation, ultimately increasing the likelihood of T cell recruitment to the tumor (23).

Axl as a dual target in cancer therapy – to suppress the hallmarks of cancer and to increase myeloid cell activation – has been underappreciated and understudied. To date, the limited number of studies acknowledging Axl and the TAMRs as dual targets have been conducted primarily in mice (20, 30–32). While these studies have demonstrated increased T cell infiltration following Axl or TAMR targeted therapy, they lack the physiological context required to gain clinically translatable mechanistic insights. Human *in vitro* studies can complement these *in vivo* findings by enabling a more thorough understanding of the myeloid cell activation required for downstream T cell recruitment and activation. Herein, we developed a human *in vitro* model system to investigate the dual effects of Axl inhibition on both tumor cells and myeloid cells in the tumor microenvironment of melanoma.

## 2 Materials and methods

### 2.1 Cell culture and reagents

The A375 human malignant melanoma cell line was purchased from ATCC (CRL-1619) and grown in DMEM (ThermoFisher 11965092) supplemented with 10% FBS (ThermoFisher 16000044), 1% Pen-Strep (ThermoFisher 15140148), and GlutaMAX (ThermoFisher 35050061). A375s were detached with 0.25% Trypsin-EDTA (ThermoFisher 25200056) and not used beyond passage 15.

Buffy coats were obtained from Massachusetts General Hospital Blood Transfusion Service for all experiments involving immune cells. Peripheral blood mononuclear cells (PBMCs) were isolated from buffy coats via density gradient centrifugation with Lymphoprep (STEMCELL 18061). PBMCs were washed twice with phosphate-buffered saline (PBS), followed by monocyte isolation with EasySep Human CD14 Positive Selection Kit II (STEMCELL 19359). Monocytes were then cultured in RPMI 1640 (ThermoFisher 32404014) supplemented with 10% heat-inactivated FBS (ThermoFisher 10082147), GlutaMAX, and HEPES (ThermoFisher 15630080). Human monocyte-derived macrophages (HMDMs) were differentiated from monocytes with 25ng/mL M-CSF (BioLegend 574804) over 6 days, with a media change on day 3. Human monocyte-derived dendritic cells (HMDDCs) were differentiated from monocytes with 20ng/mL IL-4 (Peprotech 200-04) and 40ng/mL GM-CSF (BioLegend 572903) over 8 days with a media change on days 3 and 6. Following differentiation, adherent HMDMs were washed with PBS, detached with 4mM EDTA (Corning 46-034-CI), and re-seeded in fresh media for experiments. HMDDCs, which grow in suspension, were centrifuged at 500g for 5 minutes and resuspended in fresh media prior to re-seeding for experiments. Cells were treated with 20ng/mL IFNγ (Peprotech 300-02) + 10ng/mL LPS (ThermoFisher 00-4976-93) or 20ng/mL IL-4 + 20ng/mL IL-13 (Peprotech 200-13) where stated.

All experiments were conducted in RPMI 1640 supplemented with heat-inactivated (HI) FBS, GlutaMAX, and HEPES. All cells were maintained in tissue culture-treated plastic and incubated at 37°C with 5% CO_2_.

Trametinib (S2673), dabrafenib (S2807), bemcentinib (S2841), and BMS-777607 (S1561) were purchased from SelleckChem. They were used at doses of 15nM, 150nM, 1μM, and 10μM, respectively, in all experiments. Dimethylsulfoxide (DMSO, Sigma-Aldrich D2650) was used as a dose-matched vehicle control.

### 2.2 TAMR quantification via ELISA

Cells were seeded into 6-well plates and treated for 24 hours prior to lysis. A375 lysate experiments had a density of 150,000 cells per well. HMDM and HMDDC lysate experiments had a density of 300,000 cells per well. Each well was washed once with ice-cold PBS and then lysed with 100uL of ice-cold NP40 lysis buffer (ThermoFisher J60766.AK) supplemented with protease and phosphatase inhibitors (ThermoFisher 78441). Lysates were clarified at 16,100g for 10 minutes at 4°C and stored at -80°C until further use. Total protein content in cell lysates was quantified via ThermoFisher’s Micro BCA Protein Assay Kit (23235) and was used to normalize the amount of protein added to subsequent immunoassays. R&D Systems DuoSet ELISA kits for Tyro3 (DY859), Axl (DY154), MerTK (DY6488), and Gas6 (DY885B) were used to quantify the amount of each protein in the cell lysates and, where mentioned, in the A375 supernatants.

### 2.3 Conditioned media experiments

A375s were seeded at a density of 2 million cells per T75 flask. The next day, cells were washed with PBS and then were treated with either trametinib + dabrafenib or matched DMSO vehicle control in serum-free RPMI 1640 media for 24 hours. This was followed by 24 hours of rest in serum-free RPMI 1640 media to generate injured A375s and untreated A375s, respectively. The media from these cells was then collected and clarified at 500g for 5 minutes. The supernatants were transferred to fresh tubes and diluted 1:1 with 1% HI FBS RPMI 1640 media. Diluted media was then filtered with 50mL Steriflip (MilliporeSigma SCGP00525). All other treatments in these experiments, i.e., treatments that were not conditioned media, were generated in 1:1 serum-free and 1% HI FBS RPMI 1640 media. The treatments were added to HMDMs (300,000 per well) in 6-well plates at 1mL per well and cells were incubated for 24 hours. Following this incubation, the media was collected and the HMDMs were lysed as described above. The media was clarified at 500g for 5 minutes, and the supernatant was transferred to fresh 6-well plates at 1mL per well such that donor-matched HMDDCs (300,000 per well) could be added to these wells and incubated for 24 hours. The excess HMDM supernatants were aliquoted and stored at -20°C prior to assaying via Luminex. Following the 24-hour treatment of the HMDDCs, the cells and media were collected and centrifuged at 500g for 5 minutes. The HMDDC supernatants were aliquoted and stored at -20°C for downstream analysis via Luminex while the cell pellets were washed once with PBS prior to cell lysis for downstream quantification of TAMRs via ELISA as described above.

### 2.4 Co-culture and tri-culture experiments

HMDMs were washed with PBS and treated with 1μM bemcentinib, 10μM BMS-777607, or dose-matched DMSO for 4 hours after which they were washed again with PBS. Untreated or injured A375s were generated and added to the HMDMs in their respective conditioned media as described above, and bemcentinib, BMS-777607, or DMSO was replenished. After 24 hours of co-culture, the experiments were either taken down or donor-matched HMDDCs were added to each well to begin tri-culture. Tri-culture experiments were taken down 24 hours after the addition of HMDDCs. Take down of each experiment involved collecting media supernatants, clarifying them at 500g for 5 minutes, and storing the supernatants at -20°C prior to assaying the supernatants via Luminex. Cells were collected and stained immediately to be assayed via flow cytometry. These experiments were conducted in 12-well plates with 150,000 HMDMs, 50,000 A375s, and 75,000 HMDDCs.

### 2.5 Flow cytometry

Cells were incubated with Human TruStain FcX Fc Receptor Blocking Solution (BioLegend 422302) for 30 minutes at 4°C. Cells were then washed with FACS buffer (2mM EDTA and 2% HI FBS in PBS) and stained with BV421 CD14 (BioLegend 301830), APC CD206 (BioLegend 321110), FITC CD163 (BioLegend 333618), BUV563 CD40 (BD Biosciences 741381), BV605 HLADR (BioLegend 307640), BV711 CD86 (BioLegend 305440), and PE-Cy7 CD80 (BioLegend 305218) for 20 mins at 4°C for co-culture experiments. For tri-culture experiments cells were stained with BV421 CD14, APC CD206, FITC CCR7 (BioLegend 353216), BUV563 CD40, BV605 HLADR, BV711 CD1a (BioLegend 300140), PE-Cy7 CD80, and PE CD83 (BioLegend 305308). The antibody cocktails included Brilliant Stain Buffer Plus (BD Biosciences 566385), and dilutions for each antibody are reported in **Supplementary Table 1**. Following another wash, cells were stained with Zombie NIR Fixable Viability Dye (BioLegend 423105) for 20 minutes at room temperature. The cells were then washed and fixed in 4% paraformaldehyde for 15 minutes at room temperature. Cells were washed and resuspended in FACS buffer and were maintained at 4°C prior to data acquisition. Data was acquired on a FACSymphony A3 Cell Analyzer (BD Biosciences) at the Koch Institute Flow Cytometry Core. Compensation and gating were conducted in FlowJo (10.8.2). HMDM and HMDDC fcs files were then exported from FlowJo for downstream data analysis conducted in R with the cyCombine package (33). Specifically, the data were first ArcSinh-transformed and then batch-corrected. Following batch correction, the median values for each marker for each sample were calculated, and these values were used as input for Principal Component Analysis (PCA) of HMDM and HMDDC surface marker expression.

Efferocytosis in the co-culture experiments of A375s with HMDMs was measured by staining the A375s with pHrodo Red dye (ThermoFisher P36010), which is pH sensitive, prior to starting the co-culture. HMDM consumption of A375s was determined by calculating the percent of total HMDMs in a co-culture sample that stained positively for the pHrodo Red signal.

A375 viability was measured by staining the cells with PE Annexin V (BioLegend 640947) for 10 minutes at room temperature in Annexin V Binding Buffer (BioLegend 422201). Cells were then washed and stained with Zombie Green Fixable Viability Dye (BioLegend 423111) for 10 minutes in PBS at room temperature. Following another wash, the cells were resuspended in Annexin V Binding Buffer and data was immediately acquired.

### 2.6 Luminex

Media supernatants were assayed with custom Luminex kits from R&D Systems. The list of analytes in each kit are reported in **Supplementary Table 2**. The kits were adapted to a 384-well format by using 12.5uL of sample and reagents per well. Samples were not diluted and were run in technical duplicate on a Bio-Plex 3D Suspension Array System (Bio-Rad). Data were analyzed in R. A threshold was set for each analyte based on the median fluorescent intensity (MFI) of the media background, i.e., RPMI 1640 supplemented with 0.5% heat-inactivated FBS, GlutaMAX, and HEPES. This threshold was subtracted from sample MFIs, and all negative values were set to zero. Then, these corrected MFIs were log_10_ transformed. The transformed data were analyzed with PCA, only considering analytes that were non-zero in at least 50% of the samples for conditioned media experiments or non-zero in at least 80% of the samples for co-culture and tri-culture experiments.

### 2.7 Statistical analysis

Statistical analysis was performed using GraphPad Prism 10. Bar plots show means with standard deviation as error bars. Mann-Whitney U tests were performed to compare two groups while one-way ANOVA tests, with appropriate *post-hoc* correction, were performed to compare three groups. Statistical significance was defined as follows: *p < 0.05, **p < 0.01, ***p < 0.001.

## 3 Results

### 3.1 A375 malignant melanoma cells and primary human monocyte-derived macrophages capture clinically relevant Axl behavior

We selected the A375 malignant melanoma cell line as our tumor cell model because these cells recapitulate clinically observed expression of Axl (16). Specifically, we measured A375 cell lysate expression of the TAMRs and their ligand Gas6 as well as A375 shedding or secretion of these molecules (**Figure 1A** right). These cells have no detectable MerTK, but they do express Axl, Tyro3, and Gas6 (**Figure 1B**). Notably, their levels of Axl are roughly 30x and 75x that of Tyro3 and Gas6, respectively. Furthermore, following 24-hour treatment with trametinib + dabrafenib, the MEK and BRAF inhibitors that are the standard-of-care treatment for melanoma (34), A375s increase their expression of Axl while decreasing their shedding of the receptor (**Figure 1C**). This suggests that the cells increase their Axl expression as a way to bypass the inhibitory effects of trametinib + dabrafenib on intracellular signaling (35, 36). Indeed, Axl is known to mediate drug resistance by maintaining the activity of various drug-targeted signaling pathways (15, 17, 18, 37, 38), and it is known that decreased receptor shedding is yet another avenue of drug resistance (39). Moreover, while treatment with trametinib + dabrafenib does not yield changes in A375 Gas6 expression, the cells do decrease their secretion of the ligand (**Figure 1D**). In other words, the amount of Gas6 produced by the cells remains unchanged, but the soluble amount decreases, suggesting that more Gas6 is bound to Axl on the cell surface (**Figure 1A** right) with drug treatment. This further supports the claim that A375s increase autocrine Axl signaling as a bypass mechanism to trametinib + dabrafenib treatment. Together, these results demonstrate that the A375 malignant melanoma cell line has clinically relevant responses to trametinib + dabrafenib (16) and is a viable tumor cell model to include in an *in vitro* system aimed at studying Axl inhibition.

Next, in an effort to model a wider range of potential outcomes to Axl inhibition, we generated two populations of A375s, “untreated” A375s and “injured” A375s, as the effects of Axl inhibition may differ in treatment-naïve and targeted therapy-treated tumors. Untreated A375s were generated by exposing the cells to DMSO for 24 hours followed by 24 hours of rest. Injured A375s were generated by treating the cells with trametinib + dabrafenib for 24 hours followed by 24 hours of rest. To validate that these treatments yield distinct A375 populations, we first characterized differences in the cells’ viability by measuring phosphatidylserine (PS) exposure and membrane integrity via flow cytometry. Annexin V binds to PS and is indicative of cells having undergone apoptosis, while Zombie Fixable Viability Dye stains cells with compromised cell membranes, indicative of cells having undergone necrosis. Untreated A375s are primarily live cells while injured A375s are a mix of live, apoptotic, and necrotic cells, which recapitulates differences expected between treatment-naïve and targeted therapy-treated tumors (**Figure 1E**). We also assayed the media from these A375 populations for the presence of cytokines, chemokines, and growth factors and found that untreated and injured A375s have distinct secretion profiles (**Figure 1F**). The untreated A375 secretome is rich in TGF-alpha, IL-8, and MFG-E8 while the injured A375 secretome is rich in CCL2 and S100B, indicative of a shift in molecular cues supporting tumor progression to molecular cues supporting myeloid cell recruitment (40–43). These data demonstrate that these tumor cell populations are unique in their composition and suggest that their ultimate effects on other cells in the tumor microenvironment may also be unique due to differences in signaling molecule secretion. Indeed, the motivation for including both of these populations in our work was two-fold. First, the use of the untreated A375 population allows for understanding of how treatment-naïve tumors respond to Axl inhibition, while the injured A375 population serves as a model of tumors previously treated with trametinib + dabrafenib, and is therefore clinically relevant. Second, differences observed in the secretome between treatment-naïve and targeted therapy-treated tumors mean that immune cells likely interact with these tumor cells in distinct ways, given that macrophages are extremely plastic and adapt to their microenvironment (44).

**Figure 2:**
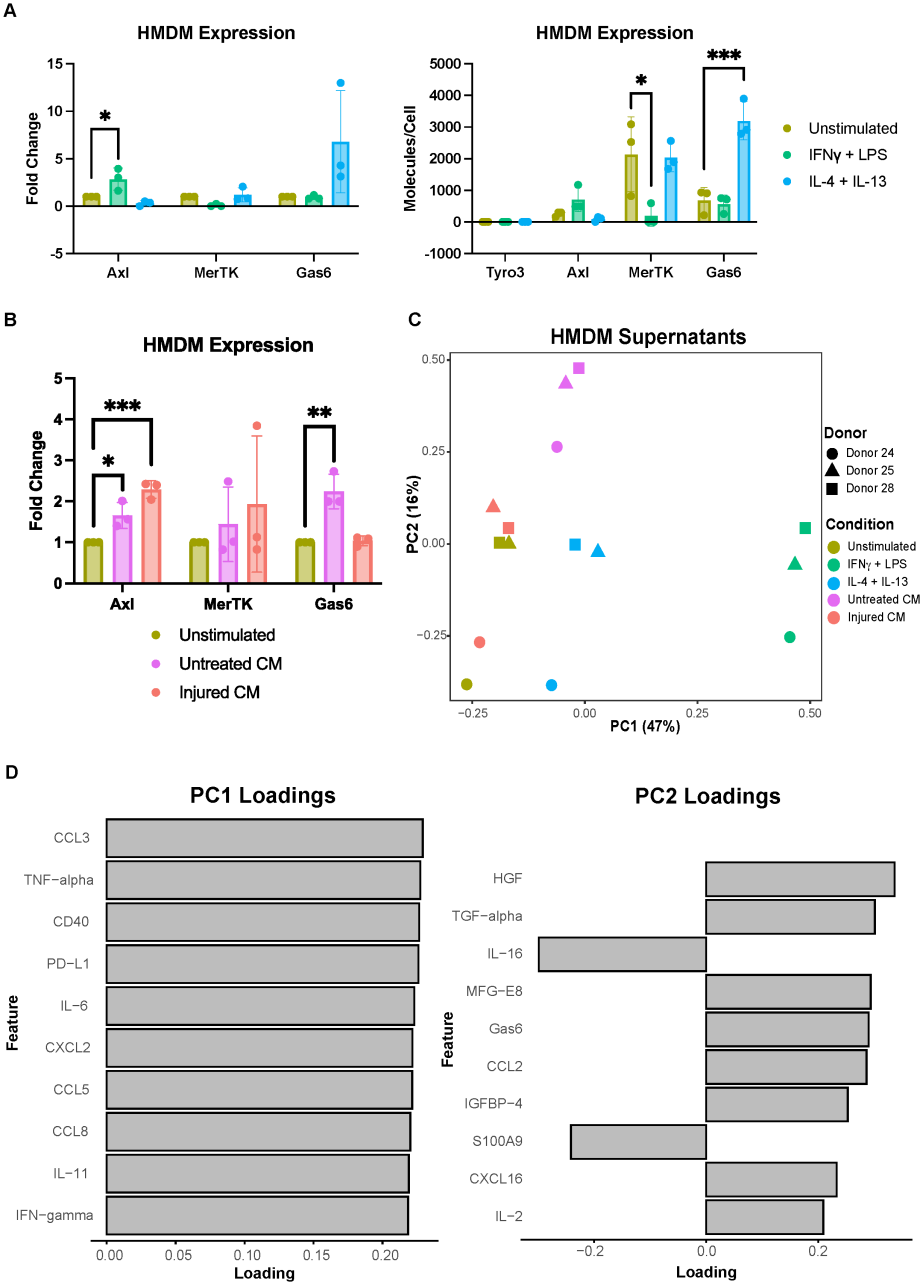
Primary human monocyte-derived macrophage expression of Axl, MerTK, and Gas6 is modulated by polarization stimuli and A375 conditioned media. **(A)** Fold change of Axl, MerTK, and Gas6 expression in polarized as compared to unstimulated HMDM cell lysates, as measured via ELISA, show IFNγ + LPS yields increased Axl expression (left). Absolute expression demonstrates decreased MerTK expression with IFNγ + LPS and increased Gas6 expression with IL-4 + IL-13 (right). Absolute expression also shows the difference in magnitude of expression of these receptors or molecules as well as the donor-to-donor variability in expression that supports the use of fold change. n = 3 independent donors. *p < 0.05, ***p < 0.001 one-way ANOVA with *post-hoc* Dunnett’s test. **(B)** Fold change of Axl, MerTK, and Gas6 expression in cell lysates of HMDMs treated with conditioned media (CM) from untreated or injured A375s as compared to unstimulated controls, measured via ELISA. Axl expression increases with both untreated and injured A375 conditioned media. Gas6 expression increases with untreated A375 conditioned media. n = 3 independent donors, same donors as **(A)**. *p < 0.05, **p < 0.01, ***p < 0.001 one-way ANOVA with *post-hoc* Dunnett’s test. **(C)** PCA scores plot of log_10_(MFI) of cytokines, chemokines, and growth factors detected via Luminex in HMDM supernatants from **(A, B)** shows separation of HMDMs treated with IFNγ + LPS, IL-4 + IL-13, and untreated A375 conditioned media. **(D)** PC1 loadings plot (left) and PC2 loadings plot (right) show which cytokines, chemokines, and growth factors contribute to separation in **(C)**. Only top 10 features shown.

Having established and characterized the A375 populations of interest, we next investigated the potential of primary human monocyte-derived macrophages (HMDMs) as the macrophage model for our *in vitro* system. We first confirmed HMDM differentiation (**Supplementary Figure 1**), and then characterized their expression of the TAMRs and Gas6 upon polarization with pro-inflammatory IFNγ + LPS or immune-resolving IL-4 + IL-13, as compared to unstimulated controls. In response to the pro-inflammatory stimuli of IFNγ + LPS, HMDMs increase their Axl expression, decrease their MerTK expression, and maintain their Gas6 expression (**Figure 2A**). Likewise, in response to the pro-resolution stimuli of IL-4 + IL-13, HMDMs slightly decrease their Axl expression, maintain their MerTK expression, and increase their Gas6 expression. Axl is considered to be the inflammatory TAMR and is known for its increased expression in inflammatory environments, while MerTK is considered to be the tolerogenic TAMR and is known to have elevated expression in homeostatic and pro-resolving environments (45). Thus, cytokine-driven changes in HMDM expression of Axl and MerTK observed here align with previously established trends, as does the HMDM expression of Gas6 (46). Notably, absolute quantification shows that Tyro3 is not detected in HMDM lysates, consistent with prior literature (47). The difference in magnitude of the absolute quantification of Axl and MerTK expression between conditions reflects their established roles as inflammatory and tolerogenic receptors, respectively. It is important to note that though donor variability is high in the absolute quantification of these molecules, the fold change in expression demonstrates consistent trends observed across donors.

While the changes in HMDM Axl, MerTK, and Gas6 expression in response to standard polarization stimuli were as expected, it was important to characterize how the distinct secreted factors of untreated and injured A375s impact this expression and, therefore, the relevance of HMDMs as the macrophage model for our *in vitro* system. Upon treatment with conditioned media from untreated or injured A375s, HMDMs universally increase their expression of Axl while changes in MerTK expression appear to be donor-dependent (**Figure 2B**). Furthermore, HMDM Gas6 expression increases with conditioned media from untreated A375s but remains unchanged with conditioned media from injured A375s. These results suggest that both the untreated A375 population and the injured A375 population train macrophages to assume a tumor-supporting role, at least in part by promoting the immunosuppressive, pro-resolution effects of macrophage Axl and MerTK signaling via signaling through their soluble factors. However, the principal component analysis (PCA) performed on Luminex results from HMDM supernatants reveal that while the macrophages that result from these two conditions exhibit comparably upregulated Axl expression, they are distinct from each other and from macrophages polarized with canonical cytokines (**Figure 2C**). Indeed, the secretion signature of HMDMs treated with IFNγ + LPS is particularly distinct from the other HMDMs (**Figure 2C**; **Supplementary Figure 2A**) because, as expected in a pro-inflammatory milieu, these macrophages secrete higher levels of most of the measured molecular cues as shown by principal component 1 (PC1) loadings (**Figure 2D**; **Supplementary Figure 2B**). Furthermore, HMDMs treated with IL-4 + IL-13 separate from those exposed to untreated A375 conditioned media along PC2, in part due to the growth factors present in media from this tumor cell population, such as TGF-alpha and MFG-E8. Surprisingly, in the PCA scores plot, HMDMs exposed to injured A375 conditioned media do not separate from their unstimulated counterparts. However, these HMDMs do separate from those exposed to untreated A375 conditioned media, demonstrating that the two tumor cell populations of interest have distinct effects on macrophage activity. Overall, these data support the use of HMDMs as an *in vitro* macrophage model to study Axl inhibition and confirm the importance of considering both untreated and injured A375 cell states.

### 3.2 Macrophage behavior is impacted by Axl inhibition and is dominated by prior tumor cell treatment

**Figure 3:**
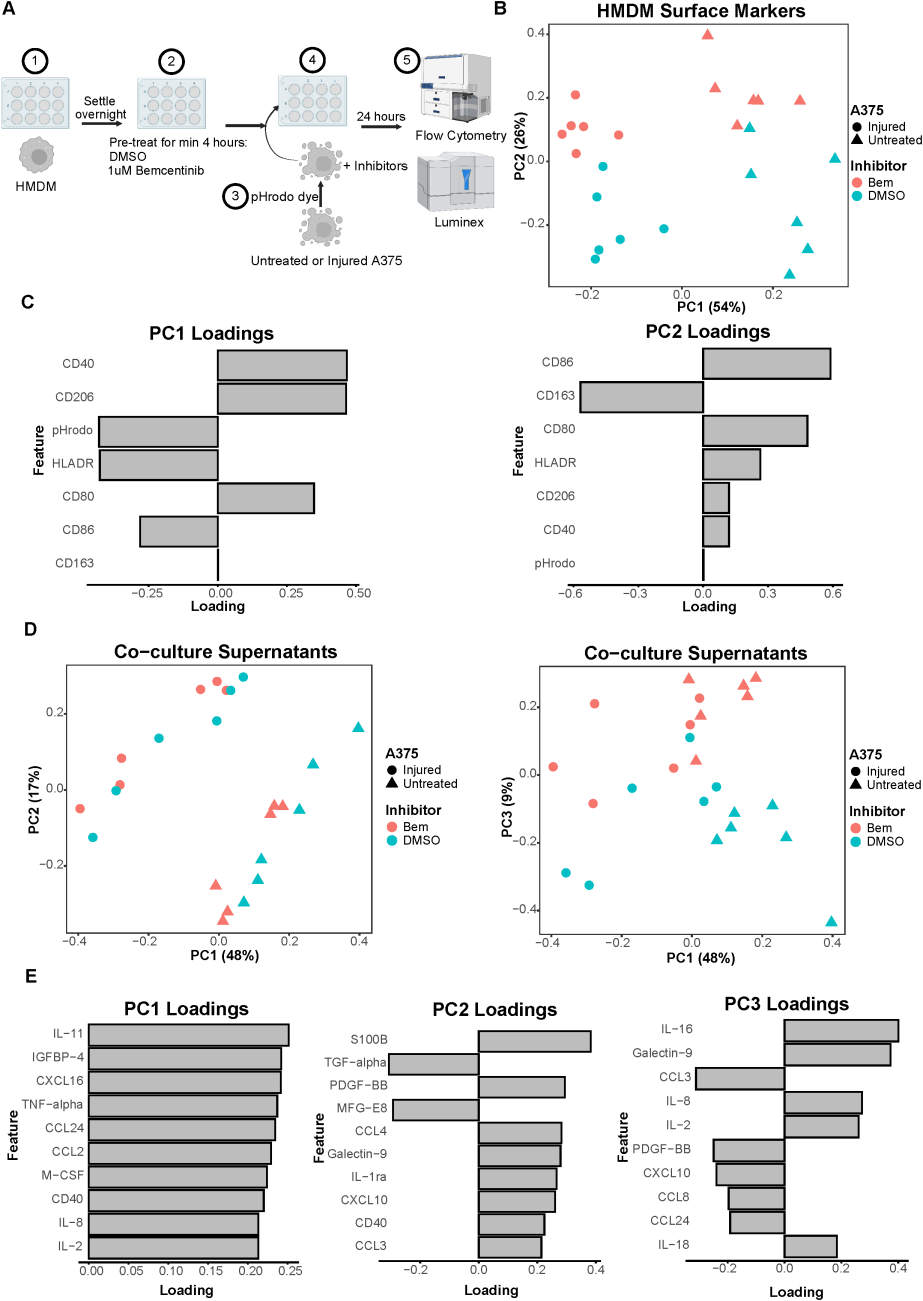
Co-culture experiments demonstrate both A375 state and inhibitor treatment contribute to macrophage state. **(A)** Schematic of experimental design: 1) After differentiation, HMDMs are harvested and re-seeded into 12-well plates. 2) HMDMs are pre-treated for at least 4 hours with 1μM bemcentinib (Bem) or DMSO vehicle. 3) During this pre-treatment, untreated and injured A375s are harvested and stained with pHrodo Red dye. 4) The pHrodo stained A375s are added to the HMDMs and the inhibitors are replenished. 5) After 24 hours of co-culture, the media is collected for downstream Luminex analysis while the cells are immediately harvested and stained for surface markers for analysis via flow cytometry. Created with BioRender.com. **(B)** PCA scores plot of surface marker expression by HMDMs in co-culture, measured via flow cytometry, shows macrophages separate based on both A375 state and inhibitor treatment. n = 6 independent donors. **(C)** PC1 loadings plot (left) and PC2 loadings plot (right) show which HMDM surface markers contribute to separation in **(B)**. **(D)** PCA scores plots of log_10_(MFI) of cytokines, chemokines, and growth factors detected via Luminex in the co-culture supernatants from **(B, C)**. PC2 vs PC1 scores plot (left) demonstrates separation of co-culture samples based on A375 state while PC3 vs PC1 scores plot (right) demonstrates separation of co-culture samples based on inhibitor treatment. **(E)** PC1 loadings plot (left), PC2 loadings plot (middle), and PC3 loadings plot (right) show which features contribute to separation in **(D)**. Only top 10 features shown.

Once our model system was established, we were first interested in how direct tumor cell-macrophage interactions may be altered by Axl inhibition. To investigate this, we co-cultured A375s with HMDMs. We pre-treated the HMDMs for at least 4 hours with either bemcentinib or DMSO vehicle prior to co-culturing them with either untreated or injured A375s (**Figure 3A**). Bemcentinib is a first-in-class Axl-specific small molecule kinase inhibitor that is in clinical trials for a variety of cancer indications, including melanoma (24), so its effects on tumor cell-macrophage interactions are clinically relevant. During this pre-treatment period, we harvested and stained untreated or injured A375s with pHrodo Red. pHrodo Red is a pH-sensitive dye that emits a strong fluorescent signal upon exposure to an acidic environment, such as the phagolysosome. Thus, the pHrodo dye enables measuring consumption of A375s by HMDMs. After staining the A375s, we added the tumor cells to the macrophages and replenished the inhibitors. Following 24 hours of co-culture, we harvested the cells and stained them with a panel of surface markers representative of immune activation state for analysis via flow cytometry. HMDMs were specifically analyzed by gating on CD14^+^ cells (**Supplementary Figure 3A**).

As many cell states are indicated by not just one surface marker but several, we again employed PCA to examine our cell populations in a multivariate manner. The PCA scores plot clearly demonstrates that the HMDMs can be distinguished by which type of A375s they were co-cultured with, i.e., untreated or injured A375s, along PC1 (**Figure 3B**). The HMDMs can also be distinguished by treatment with bemcentinib or DMSO along PC2. Importantly, the separation along these PCs suggests that the A375 state dominates macrophage behavior because PC1 captures the greatest variance in the data, and this variance is defined by the tumor cell state. In order to identify markers driving the separation by A375 state and inhibitor treatment, we looked at the feature loadings contributing to each PC. The PC1 loadings (**Figure 3C**) indicate that the pHrodo dye signal is higher in HMDMs co-cultured with injured A375s, which is consistent with the consumption data from these experiments (**Supplementary Figure 3B**). CD86 and HLADR also contribute to the separation of these macrophages from those co-cultured with untreated A375s, suggesting that injured A375s promote HMDMs to be better equipped for antigen presentation. In contrast, HMDMs co-cultured with untreated A375s are associated with expression of CD40, CD80, and CD206, meaning that these macrophages have adopted a complex tumor-associated macrophage state and, if given the appropriate cues, may be manipulated into an anti-tumor state. With respect to separation along PC2, CD86, CD80, HLADR, CD40, and CD206 expression is in the direction of bemcentinib treatment, while CD163 expression is in the direction of DMSO vehicle treatment (**Figure 3C**). The HMDM surface marker expression profile with bemcentinib treatment suggests that these macrophages are primarily in an antigen-presenting, pro-inflammatory, and anti-tumor state. We had hypothesized that Axl inhibition via bemcentinib treatment would yield decreases in macrophage consumption of A375s, given that Axl is an efferocytic receptor. However, the pHrodo dye signal does not associate with either direction of separation along PC2, and upon further analysis, we found that this is consistent with the consumption data from these experiments (**Supplementary Figure 3C**). Bemcentinib’s lack of impact on HMDM consumption of A375s may be explained in part by a compensatory increase in HMDM total MerTK expression (**Supplementary Figure 4**) as well as by other phagocytic receptors’ signaling remaining intact.

While HMDM surface marker expression hints at macrophage state, it is insufficient to understand macrophage activity. In an effort to understand differences in macrophage activity under these various co-culture conditions, we collected media supernatant from each sample to assess changes in molecular cues via Luminex (**Supplementary Figure 5A**). Though secreted cues in supernatant could be contributed by either HMDMs or A375s in this co-culture experiment, it is informative of the overarching immune microenvironment that the HMDMs are exposed to. We found that, like in the HMDM flow cytometry data, the supernatants of the co-culture samples separate primarily based on A375 state, i.e., untreated or injured, as depicted in the PCA scores plot of PC2 against PC1 (**Figure 3D**). However, the multivariate effects of the inhibitors, i.e., bemcentinib or DMSO vehicle, are not clear, in part due to donor-to-donor variability (**Supplementary Figure 5B**). When examining the combination of PC1 and PC3, we found PC3 is able to separate the samples based on inhibitor treatment (**Figure 3D**). As these differences are seen on a higher principal component, this suggests that A375 state again influences the secretome to a greater degree than the inhibitor treatment. To identify which cytokines, chemokines, and growth factors are contributing to these observations, we turned to the feature loadings of each PC. The PC1 loadings plot demonstrates that the top features are highly present in samples with untreated A375s (**Figure 3E**). In fact, nearly all of the features are present at higher levels with untreated A375s (**Supplementary Figure 5C**), reinforcing the claim that the molecular cues in the microenvironment of a treatment-naïve tumor are distinct from those of a targeted therapy-treated tumor. The PC2 loadings plot describes donor differences in secreted molecular cues (**Figure 3E**; **Supplementary Figure 5C**), which is consistent with the high level of variability known to exist between human macrophages (48). Finally, the PC3 loadings plot identifies high IL-16, Galectin-9, IL-8, IL-2, and IL-18 as the features associated with bemcentinib treatment while high CCL3, PDGF-BB, CXCL10, CCL8, and CCL24 are associated with DMSO vehicle (**Figure 3E**). Though these results indicate that the molecular cues secreted under bemcentinib treatment differ from those secreted under DMSO vehicle treatment, neither set of cues is a clear, cohesive pro-inflammatory, anti-tumor signature, suggesting that the signaling changes induced by bemcentinib are pleiotropic and will likely influence the immune microenvironment in a context-dependent manner.

### 3.3 Primary human monocyte-derived dendritic cells dampen the effects of Axl inhibition

We have thus far focused on HMDMs because macrophages are the most abundant immune cell in solid tumors (49), but macrophages are not the only myeloid cell potentially impacted by Axl inhibition, as dendritic cells are also known to rely on TAMR signaling (25). Given that these myeloid cells are crucial to establishing a durable anti-tumor immune response through T cell activation, we next incorporated human monocyte-derived dendritic cells (HMDDCs) into our *in vitro* system.

**Figure 4:**
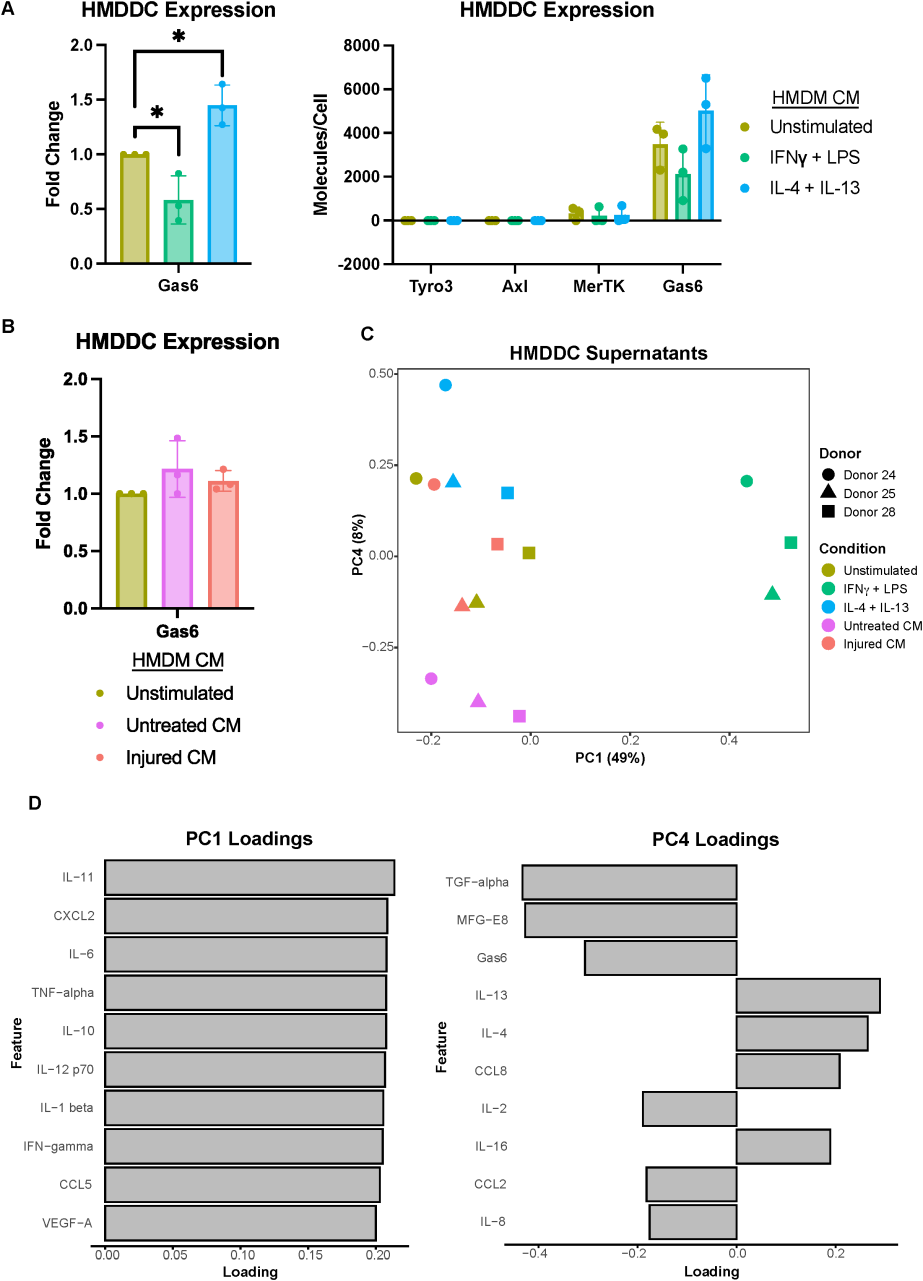
Primary human monocyte-derived dendritic cell expression of Gas6 is modulated by polarization stimuli but not A375 secreted factors. **(A)** Fold change of Gas6 expression in polarized as compared to unstimulated HMDDC cell lysates, as measured via ELISA, demonstrates a decrease in a decrease in expression with IFNγ + LPS and an increase with IL-4 + IL-13 (left). Conditioned media (CM) treatments are from donor-matched HMDMs. Absolute expression demonstrates the general lack of TAMR expression by HMDDCs (right). n = 3 independent donors, same donors as **Figure 2**. *p < 0.05 one-way ANOVA with *post-hoc* Dunnett’s test. **(B)** Fold change of Gas6 expression in cell lysates of HMDDCs treated with conditioned media from untreated or injured A375s as compared to unstimulated controls, measured via ELISA. A375 secreted factors do not affect dendritic cell Gas6 expression. Conditioned media are from donor-matched HMDMs. n = 3 independent donors, same donors as **(A)** and **Figure 2**. **(C)** PCA scores plot of log_10_(MFI) of cytokines, chemokines, and growth factors detected via Luminex in HMDDC supernatants from **(A, B)** shows separation of HMDDCs treated with IFNγ + LPS, IL-4 + IL-13, and untreated A375 conditioned media. **(D)** PC1 loadings plot (left) and PC4 loadings plot (right) show which cytokines, chemokines, and growth factors drive separation in **(C)**. Only top 10 features shown.

We first confirmed HMDDC differentiation (**Supplementary Figure 1**), and then characterized the TAMR and Gas6 expression of these cells. We found that while they do not express Tyro3 or Axl, they do express low levels of MerTK (**Figure 4A**). Despite their general lack of TAMR expression, HMDDCs express copious amounts of the ligand Gas6 (**Figure 4A** right), as compared to HMDMs (**Figure 2A** right), and this expression is modulated by polarization stimuli. Interestingly, unlike their HMDM counterparts (**Figure 2B**), HMDDC expression of Gas6 is not influenced by exposure to untreated or injured A375 conditioned media (**Figure 4B**). While the cells’ Gas6 expression remained unchanged, we hypothesized that HMDDCs may alter other secreted factors in response to treatment with polarization stimuli or conditioned media. To investigate this, we assayed their media supernatant via Luminex (**Supplementary Figure 6A**). As expected, dimensionality reduction separates the IFNγ + LPS treated HMDDCs from all others along PC1 (**Figure 4C**) because these polarization stimuli promote increased secretion of nearly all of the molecular cues measured (**Figure 4D**; **Supplementary Figure 6C**). Separation along PC2 and PC3 is dominated by donor differences (**Supplementary Figure 6B**). These donor differences are also evident in PC2 of the donor-matched HMDMs (**Figure 2C**) from which media was sourced to treat the HMDDCs, but the donor differences do not dominate variance along PC2 of the macrophage supernatants. We looked for principal components that were able to cluster donors together but separate HMDDCs based on treatment condition, and found that this was true of PC4. Specifically, the dendritic cells exposed to IL-4 + IL-13 conditioned media separate from those exposed to untreated A375 conditioned media (**Figure 4C**). The feature loadings for PC4 (**Figure 4D**) indicate that this separation is driven by some factors that are present in either the untreated A375 conditioned media (**Figure 1F**) or the IL-4 + IL-13 HMDM supernatants (**Figure 2D**). Overall, these data demonstrate that the HMDDCs can be distinguished by the media they are treated with.

**Figure 5:**
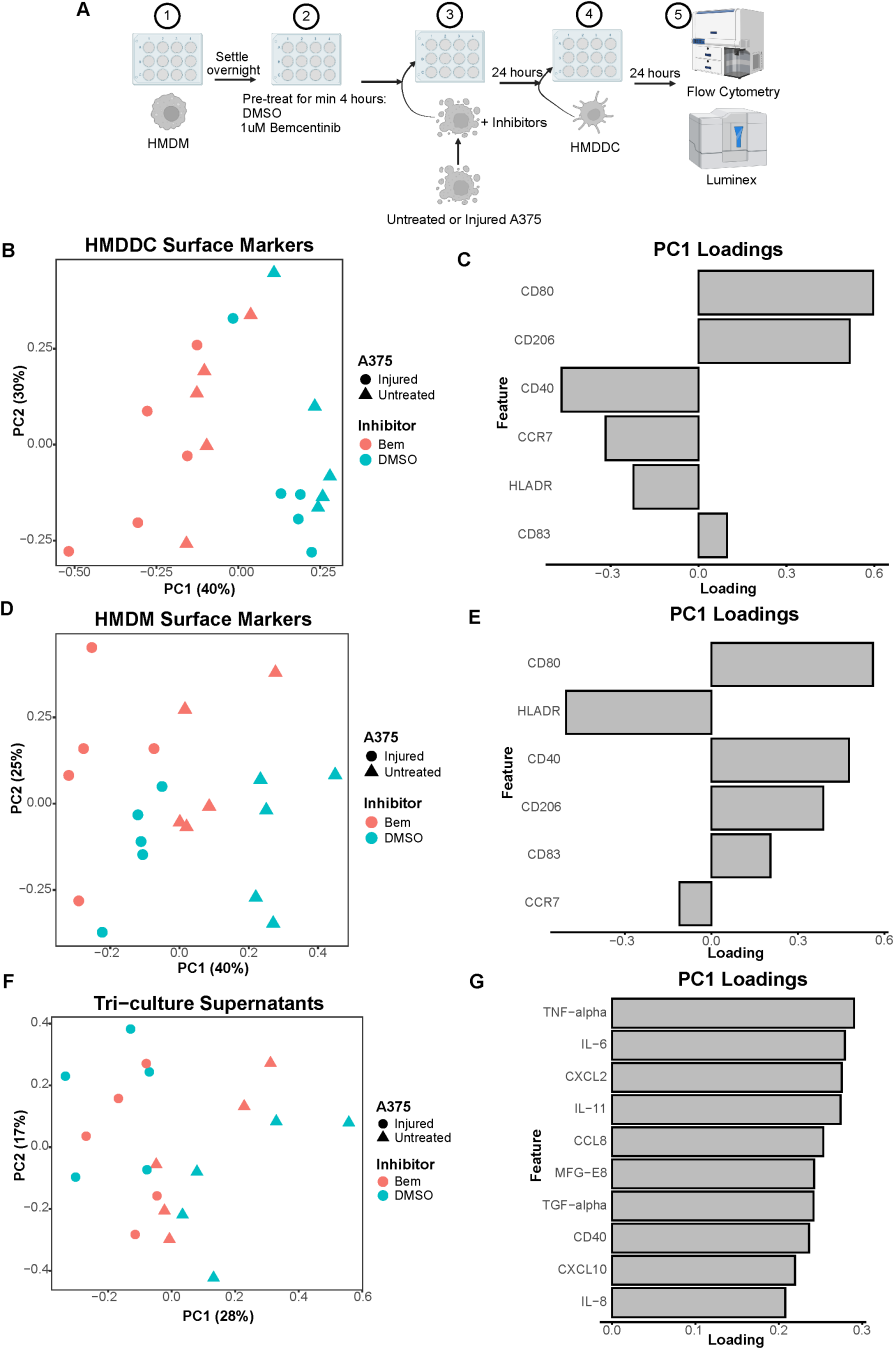
The addition of HMDDCs to the system serves as a perturbation that dampens macrophage activity. **(A)** Schematic of experimental design: 1) After differentiation, HMDMs are harvested and re-seeded into 12-well plates. 2) HMDMs are pre-treated for at least 4 hours with 1μM bemcentinib (Bem) or DMSO vehicle. 3) During this pre-treatment, untreated and injured A375s are harvested and are then added to the HMDMs, and the inhibitors are replenished. 4) After 24 hours of co-culture, donor-matched HMDDCs are added. 5) After 24 hours of tri-culture, the media is collected for downstream Luminex analysis while the cells are immediately harvested and stained for surface markers for analysis via flow cytometry. Created with BioRender.com. **(B)** PCA scores plot of surface marker expression by HMDDCs in tri-culture, measured via flow cytometry, shows dendritic cells separate primarily based on inhibitor treatment, and within inhibitor treatment arguably by A375 state. n = 5 independent donors. **(C)** PC1 loadings plot shows which HMDDC surface markers contribute to separation in **(B)**. **(D)** PCA scores plot of surface marker expression by HMDMs in tri-culture, measured via flow cytometry, shows macrophages separate primarily based on A375 state, and potentially separate further by inhibitor treatment. n = 5 independent donors, same donors as **(B)**. **(E)** PC1 loadings plot shows which HMDM surface markers contribute to separation in **(D)**. **(F)** PCA scores plot of log_10_(MFI) of cytokines, chemokines, and growth factors detected via Luminex in the tri-culture supernatants from **(B)** through **(E)**. Tri-cultures separate by A375 state. **(G)** PC1 loadings plot shows which features contribute to separation in **(F)**. Only top 10 features shown.

To better understand the direct effects of the A375s and HMDMs on the dendritic cells as well as the effects of the HMDDCs – and their copious amounts of Gas6 – on the system, we added the dendritic cells to our co-culture experiments to conduct tri-culture experiments. First, the co-culture was conducted as discussed in the previous section, with the exception that the A375s were not stained with pHrodo Red (**Figure 5A**). Once the 24-hour co-culture of HMDMs with A375s was completed, donor-matched HMDDCs were added for 24 hours of tri-culture prior to sample collection. Importantly, the HMDDCs were added after the 24-hour co-culture of A375s and HMDMs in an effort to model dendritic cell infiltration that may result from changes to the immune microenvironment following treatment with bemcentinib.

We again utilized a panel of cell surface markers that enabled us to distinguish our cell populations and characterize immune cell activation state via flow cytometry. We were able to distinguish HMDDCs from HMDMs based on their expression of CD1a or CD14, respectively (**Supplementary Figures 1C, D**). Following gating on CD1a^+^CD14^-^ cells to exclusively characterize HMDDCs, we performed PCA on the batch-corrected surface marker expression data. Separation along PC1 is dominated by treatment with bemcentinib or DMSO vehicle (**Figure 5B**). Arguably, within each inhibitor, HMDDCs display some degree of separation based on A375 state, i.e., untreated or injured. The feature loadings for PC1 indicate that HMDDC expression of HLADR, CD40, and CCR7 is associated with bemcentinib treatment while expression of CD80, CD83, and CD206 is associated with DMSO vehicle treatment (**Figure 5C**). These data suggest that regardless of A375 state, bemcentinib promotes HMDDCs to adopt a more mature state.

Not only can we interrogate changes to HMDDC surface marker expression in the tri-culture experiments, but we can also assess changes to HMDM surface marker expression in these experiments. After gating on CD1a^-^CD14^+^ cells to exclusively characterize HMDMs in the system, PCA of the batch-corrected surface marker expression data shows that the macrophages separate by A375 state along PC1 (**Figure 5D**). This separation is driven by CD80, CD83, CD40, and CD206 expression in the direction of tri-culture with untreated A375s (**Figure 5E**), further supporting the claim that HMDMs cultured with the untreated A375 population assume a complex tumor-associated macrophage state while those cultured with the injured A375 population may be better equipped for antigen presentation. Though it could be argued that within each A375 cell state there is separation based on whether the tri-culture was treated with bemcentinib or DMSO vehicle, the macrophages in tri-culture do not separate based on both A375 state and inhibitor treatment as cleanly as the macrophages in the co-culture experiments do (**Figure 3B**). This suggests that the addition of dendritic cells is a significant perturbation to the system. Indeed, the expression of CD206 and CD40 decreases in HMDMs treated with the stimuli of IFNγ + LPS or IL-4 + IL-13, and even in HMDMs cultured with untreated A375s, following the addition of HMDDCs (**Supplementary Figure 7**). This dampened macrophage response indicates that macrophages adapt to the presence of dendritic cells in the system, and these immune cells likely coordinate to share the burden of responding to the tumor cells in the system.

As in the co-culture experiments, surface marker expression is informative of macrophage and dendritic cell state but is insufficient to understand changes to the immune microenvironment. So, we collected media supernatants from the tri-culture samples to examine differences in the molecular cues of the various conditions via Luminex (**Supplementary Figure 8A**). We found that the tri-culture samples primarily separate based on whether the cultures included untreated or injured A375s along PC1 (**Figure 5F**), and this is driven by greater secretion in samples containing the untreated A375 population (**Figure 5G**; **Supplementary Figure 8B**). The observation that most of the molecular cues measured are more highly present in tri-culture samples with untreated A375s is in agreement with the co-culture supernatant data (**Figures 3D, E**; **Supplementary Figure 5C**), further emphasizing the impact of tumor cell prior treatment status on the immune microenvironment. In contrast to the co-culture supernatant data, the molecular cues present in the tri-culture supernatants do not clearly separate with bemcentinib or DMSO vehicle treatment. This lack of separation indicates that the addition of HMDDCs to the system may hinder the effects of bemcentinib and further supports the claims that the dendritic cells act as a perturbation to the system and dampen macrophage response to the inhibitor.

### 3.4 Axl inhibition and pan-TAMR inhibition are not interchangeable

**Figure 6:**
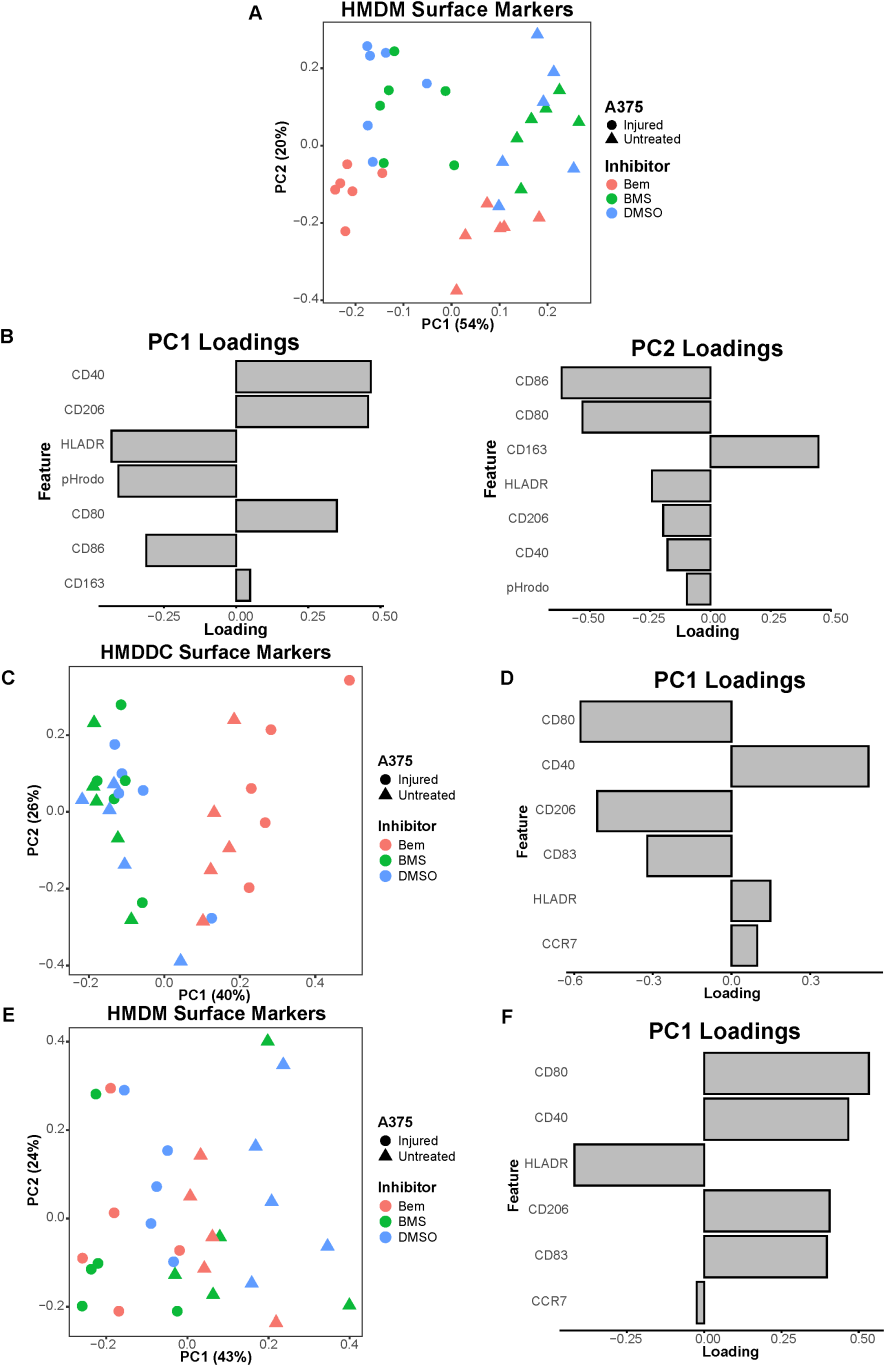
The pan-TAMR small molecule inhibitor BMS-777607 does not recapitulate the results observed with bemcentinib in this *in vitro* system. **(A)** PCA scores plot of surface marker expression by HMDMs in co-culture, measured via flow cytometry, shows macrophages separate based on both A375 state and inhibitor treatment. BMS-777607 (BMS) treatment does not separate from DMSO vehicle treatment while bemcentinib (Bem) treatment does. n = 6 independent donors, same donors as **Figure 3**. **(B)** PC1 loadings plot (left) and PC2 loadings plot (right) show which HMDM surface markers contribute to separation in **(A)**. **(C)** PCA scores plot of surface marker expression by HMDDCs in tri-culture, measured via flow cytometry, distinguishes dendritic cells in tri-cultures treated with bemcentinib from those treated with DMSO vehicle or BMS-777607. n = 5 independent donors, same donors as **Figure 5**. **(D)** PC1 loadings plot shows which HMDDC surface markers contribute to separation in **(C)**. **(E)** PCA scores plot of surface marker expression by HMDMs in tri-culture, measured via flow cytometry, shows macrophages separate based on A375 state. n = 5 independent donors, same donors as **(C)** and **Figure 5**. **(F)** PC1 loadings plot shows which HMDM surface markers contribute to separation in **(E)**.

Recognizing that Axl is not the only TAMR expressed by myeloid cells and is not the sole TAMR target under clinical investigation (24, 50), we conducted these same experiments with the pan-TAMR inhibitor BMS-777607. It should be noted that we selected the dose of this inhibitor based on previous literature demonstrating that a dose of 10μM results in 77% inhibition of efferocytosis *in vitro* (20). Interestingly, though BMS-777607 decreases HMDM consumption of injured A375s compared to DMSO vehicle control in the co-culture experiments (**Supplementary Figure 9**), this functional difference is not reflected by changes in HMDM surface marker expression, as these macrophages cluster with the DMSO-treated condition (**Figure 6A**). The macrophages co-cultured with the untreated A375 population adopt a complex tumor-associated macrophage state (**Figure 6B**, PC1 loadings) and are distinguishable from those co-cultured with the injured A375 population, as expected. However, treatment with BMS-777607 does not promote the antigen-presenting, pro-inflammatory, and anti-tumor macrophage state observed with bemcentinib treatment (**Figure 6B**, PC2 loadings). Furthermore, in the tri-culture system, dendritic cells from samples treated with BMS-777607 do not separate from those treated with DMSO vehicle (**Figure 6C**), and they do not adopt the more mature state observed with bemcentinib treatment (**Figure 6D**). Consistent with the previously discussed data, the macrophages in the tri-culture system separate based on whether or not the A375 cells were pre-treated with targeted therapy, but not based on TAMR inhibitor treatment (**Figure 6E**). Furthermore, the HMDMs from tri-culture with untreated A375s adopt a complex tumor-associated macrophage state (**Figure 6F**). Taken together, these data demonstrate that Axl inhibition is not interchangeable with pan-TAMR inhibition, at least in this *in vitro* system.

## 4 Discussion

**Figure 7:**
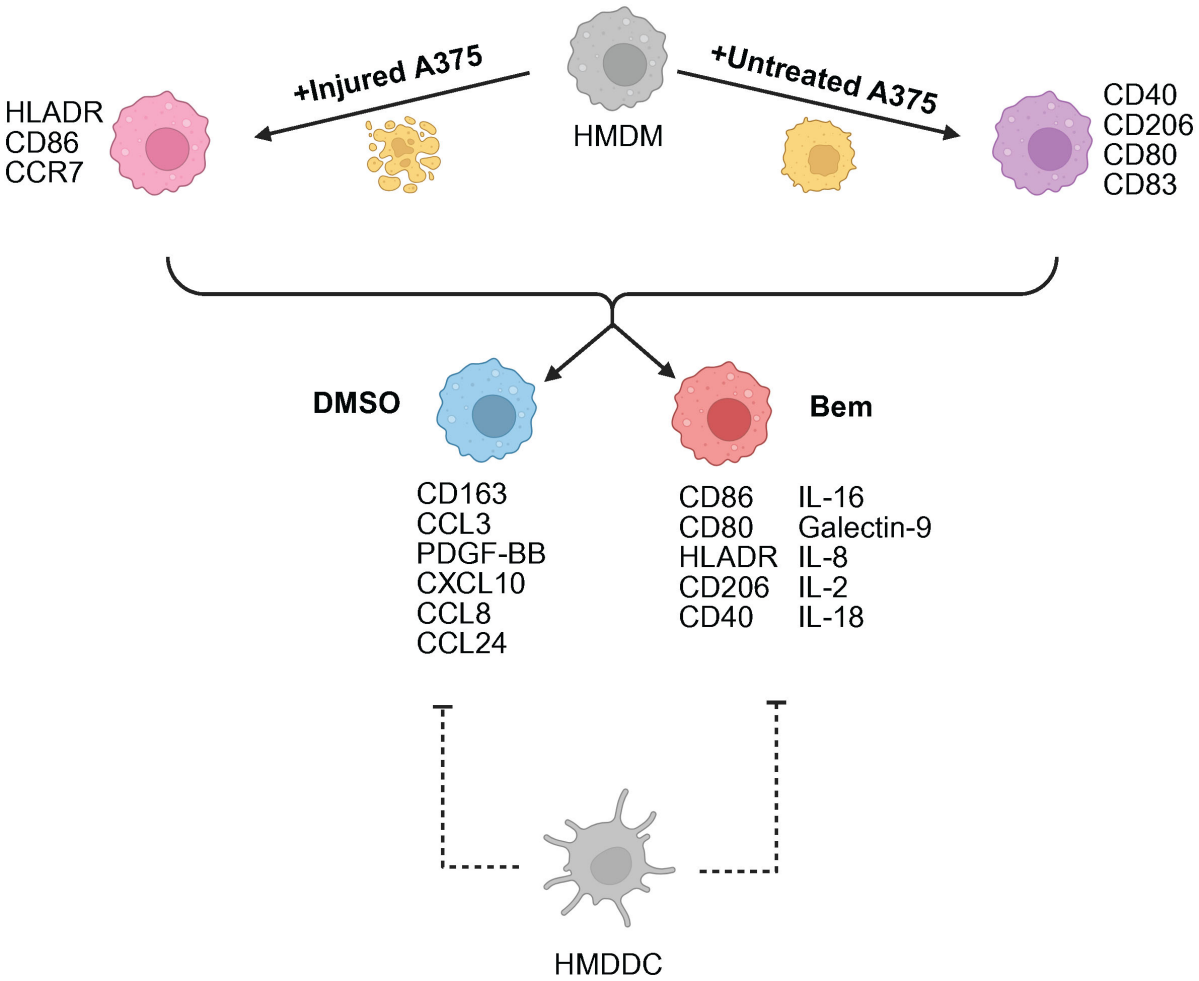
Summary schematic of insights gained from this study. A375 prior treatment with targeted therapy is the primary driver of macrophage (HMDM) state. Axl inhibition via bemcentinib (Bem) promotes macrophage activation and antigen presentation, characterized by CD86, CD80, HLADR, and CD40 expression. The addition of dendritic cells (HMDDC) to the system dampens macrophage response. Created with BioRender.com.

In this work, we developed a human *in vitro* model system of the tumor microenvironment to study the effects of Axl inhibition on myeloid cells. We found that the Axl-specific small molecule inhibitor bemcentinib promotes increased activation and antigen presentation by HMDMs and HMDDCs. This suggests that bemcentinib primes the myeloid compartment of the tumor microenvironment to be better equipped to support increased T cell infiltration into the tumor for a durable anti-tumor immune response, as has been observed in prior *in vivo* studies (30). Notably, through our experiments comparing bemcentinib to the pan-TAMR inhibitor BMS-777607 we demonstrate that TAMR inhibition is not necessarily interchangeable with Axl inhibition. However, more comprehensive studies are warranted to understand whether this finding extends to other forms of TAMR inhibition to uncover the potential differential effects of Axl inhibition and pan-TAMR inhibition, ultimately furthering the field’s understanding of the roles of Tyro3, Axl, and MerTK in the tumor microenvironment. Finally, due to the design control inherent to *in vitro* experiments, we were able to consider tumor cell state, i.e., untreated vs. injured through treatment with targeted therapeutics, as a variable necessary to gain a mechanistic understanding of Axl inhibition in the distinct contexts of a treatment-naïve tumor microenvironment versus a tumor microenvironment defined by prior standard-of-care treatment (**Figure 7**) – a comparison which, to the best of our knowledge, has not been reported on previously.

It is well-known that the form of death incurred by a cell as well as its associated molecular cues impact macrophage response and behavior upon efferocytosing the dead cell, as detailed by Rothlin et al. (51). Recently, it was also shown that the identity of the dead cell it consumes ultimately impacts macrophage response to exogenous stimuli (52). Thus, we hypothesized that injury via treatment with targeted therapy may impact subsequent macrophage response to tumor cells, and that the distinct roles assumed by these macrophages may fundamentally alter their responses to Axl inhibition. Strikingly, untreated A375s yield HMDMs exhibiting a tumor-associated macrophage phenotype characterized by CD40, CD80, and CD206 surface expression while injured A375s yield HMDMs with markers of increased antigen presentation, namely HLADR and CD86. These A375 populations also resulted in co-cultures with distinct molecular signatures, where the majority of the measured cues were found to be more highly secreted in co-cultures with untreated A375s. It is important to note that measuring cytokines, chemokines, and growth factors in media supernatants is limited to detecting those that are produced in excess. In other words, molecules that are produced and consumed or degraded at relatively equivalent rates cannot be detected. Thus, while one interpretation of our Luminex data is that the microenvironment of HMDMs cultured with untreated A375s is marked by higher immune activity, it is also possible that the decreased presence of these molecular cues in the injured A375 co-cultures is because the molecules are consumed at a rate faster than they are produced as a direct result of increased signaling and immune activity. Another possible explanation of the lower amounts of secreted proteins detected in supernatants from co-cultures with injured A375s may simply be that these injured tumor cells do not produce the secreted factors at appreciable levels. Regardless of the interpretation of the Luminex data, it is clear that tumor cell state is an important variable to consider that yields distinct macrophages and microenvironments. Importantly, these differences may ultimately impact the effects of Axl inhibition. Indeed, even though bemcentinib treatment consistently separates from DMSO vehicle control in our co-culture data, within the bemcentinib treatment the conditions remain separate based on tumor cell state, further supporting the hypothesis that the immune cell response to Axl inhibition is impacted by the perturbed tumor state that results from prior treatment with other therapeutics. This hypothesis warrants further investigation because it has important implications for the clinical use of Axl inhibitors and their integration into standard-of-care frontline therapies for cancer patients.

For use in combination with existing treatments, our work suggests that treatment timing could affect the immune activation landscape with potential implications on efficacy. Indeed, it has been demonstrated that following the development of treatment resistance to chemotherapy, the use of bemcentinib as a monotherapy is insufficient to prolong survival in a preclinical study of leukemia (32). More recent results do show that in acute myeloid leukemia, the combination of bemcentinib with low dose chemotherapy is beneficial in a subset of patients (53). In the context of non-small cell lung cancer (NSCLC), a recent study demonstrated that Axl expression in the tumor and immune compartments was not associated with overall survival in patients who received first line immunotherapy, but in patients who had received prior chemotherapy, Axl expression was a strong negative prognostic factor (54). Congruently, a clinical trial (NCT03184571) assessing bemcentinib in combination with anti-PD-1 immunotherapy showed that Axl-expressing NSCLC patients have improved response (55). Additionally, administration of bemcentinib benefitted a subset of EGFR mutant patients who had failed prior erlotinib targeted therapy (NCT02424617, 56). However, clinical trials in the first line setting have shown mixed results. Bemcentinib in the context of first line metastatic melanoma treatment (NCT02872259) did not increase patient response or survival (57), and a first line combination treatment trial in STK11-mutant NSCLC was recently discontinued (NCT05469178, 58). Other Axl targeting agents (e.g., sitravatinib) have also reported disappointing clinical trial results (NCT03906071, 59). Therefore, there is a need to elucidate the context-specific mechanistic effects of Axl inhibition on the tumor microenvironment, as validated by our *in vitro* model. Additional models such as clinical solid tumor samples could provide insights into a more complex tumor microenvironment that extend beyond our three cell types and broaden the clinical relevance of our findings. To that end, following the publication of clinical trial data, it will be imperative to assess the ability of this *in vitro* model to capture clinical observations.

Given our interest in how Axl inhibition may alter the tumor immune microenvironment to ultimately support T cell infiltration and an anti-tumor immune response, we conducted tri-culture experiments with HMDDCs. Like the macrophages in the co-culture experiments, we found that the dendritic cells in tri-culture separate based on inhibitor treatment, with bemcentinib treatment being associated with increased expression of maturation markers. Intriguingly, including HMDDCs in the system appears to dampen the HMDM responses that were observed in the co-culture experiments. Specifically, the addition of HMDDCs impedes the inhibitor-associated separation of HMDMs as assessed by surface marker expression, and it impedes the inhibitor-associated separation of the secreted molecular cues as assessed by Luminex. We hypothesize that adding HMDDCs to the previously established co-culture system is a significant perturbation to macrophage signaling, and that the observed modulation of HMDM response may be explained in part by the macrophages and dendritic cells cooperating to share the burden of the innate immune response to the tumor cells. Indeed, though macrophages and dendritic cells are traditionally considered to have disparate roles in the immune system, the field has begun to recognize that these cells cooperate and coordinate their functions (60). Therefore, future studies pursuing this hypothesis are warranted. Notably, despite the observation that HMDDCs dampen the effects of bemcentinib on HMDM state and on the molecular signature of the system, the effects of tumor cell pre-treatment with targeted therapy are maintained. This further bolsters the claim that treatment-naïve and targeted therapy-treated tumor cells likely predetermine the therapeutic efficacy of Axl inhibition as a consequence of how they condition the immune cells in their microenvironment. That is, the tumor cell state may dictate the nature of the immune cell response to Axl inhibition, and future studies should explore this possibility.

In conclusion, we have demonstrated the importance of investigating the effects of Axl inhibition on myeloid cells in addition to tumor cells in the tumor microenvironment. We have shown that the effects of Axl inhibition depend not only on the cellular composition of the model system, but also on the previous treatment status of the tumor cells. Finally, we have reinforced the utility of human *in vitro* model systems in complementing insights gained from *in vivo* studies. Specifically, these *in vitro* models contribute to the elucidation of the complex mechanistic effects of candidate therapies, as they can be used to investigate cell-cell communication and changes to immune cell crosstalk that may impact T cell recruitment and activation. Collectively, these models have the potential to inform clinical trial design by evaluating efficacy in an immune landscape defined by standard-of-care treatments.

## Data Availability

The raw data supporting the conclusions of this article will be made available by the authors, without undue reservation.
