# Supplementary material for: Axl inhibitor-mediated reprogramming of the myeloid compartment of the in vitro tumor microenvironment is influenced by prior targeted therapy treatment
Source: Front Immunol. 2025 Jun 5;16:1601420. doi: 10.3389/fimmu.2025.1601420 (PMC12176863; doi:10.3389/fimmu.2025.1601420)
Supplement: Supplementary file 1 [file DataSheet1.docx]

**Supplementary Table 1.** Dilutions used for staining co-culture and tri-culture samples.

| **Antibody** | **Clone** | **Catalog Number** | **Dilution** |
| --- | --- | --- | --- |
| BV421 CD14 | M5E2 | BioLegend 301830 | 100x |
| APC CD206 | 15-2 | BioLegend 321110 | 200x |
| FITC CD163 | GHI/61 | BioLegend 333618 | 200x |
| BUV563 CD40 | 5C3 | BD Biosciences 741381 | 200x |
| BV605 HLADR | L243 | BioLegend 307640 | 100x |
| BV711 CD86 | IT2.2 | BioLegend 305440 | 100x |
| PE-Cy7 CD80 | 2D10 | BioLegend 305218 | 100x |
| FITC CCR7 | G043H7 | BioLegend 353216 | 100x |
| BV711 CD1a | HI149 | BioLegend 300140 | 100x |
| PE CD83 | HB15e | BioLegend 305308 | 100x |

**Supplementary Table 2.** Full list of the 43 analytes included in custom kits for assaying media supernatants via Luminex. The italicized analytes (EGF and PDGF-CC) were not included in all experiments. EGF was included but PDGF-CC was not in the 43-plex custom Luminex kits used to assay media in Figures 1, 2, and 4. EGF was not included while PDGF-CC was included in the 43-plex custom Luminex kits used to assay media in Figures 3 and 5.

| Custom 23-Plex Kit | | | |
| --- | --- | --- | --- |
| CCL2 | CCL3 | CCL4 | CCL13 |
| CCL18 | CCL23 | CCL24 | CD40 |
| CD40 Ligand | CXCL2 | CXCL10 | CXCL16 |
| Galectin-9 | Gas6 | HGF | IFN-gamma |
| IL-1 beta | IL-6 | IL-10 | IL-18 |
| PD-L1 | TNF-alpha | VEGF-A | *EGF* |
| Custom 20-Plex Kit | | | |
| CCL5 | CCL8 | GM-CSF | IGFBP-4 |
| IL-1 alpha | IL-1ra | IL-2 | IL-4 |
| IL-8 | IL-11 | IL-12 p70 | IL-13 |
| IL-16 | M-CSF | MFG-E8 | PDGF-BB |
| *PDGF-CC* | S100A9 | S100B | TGF-alpha |


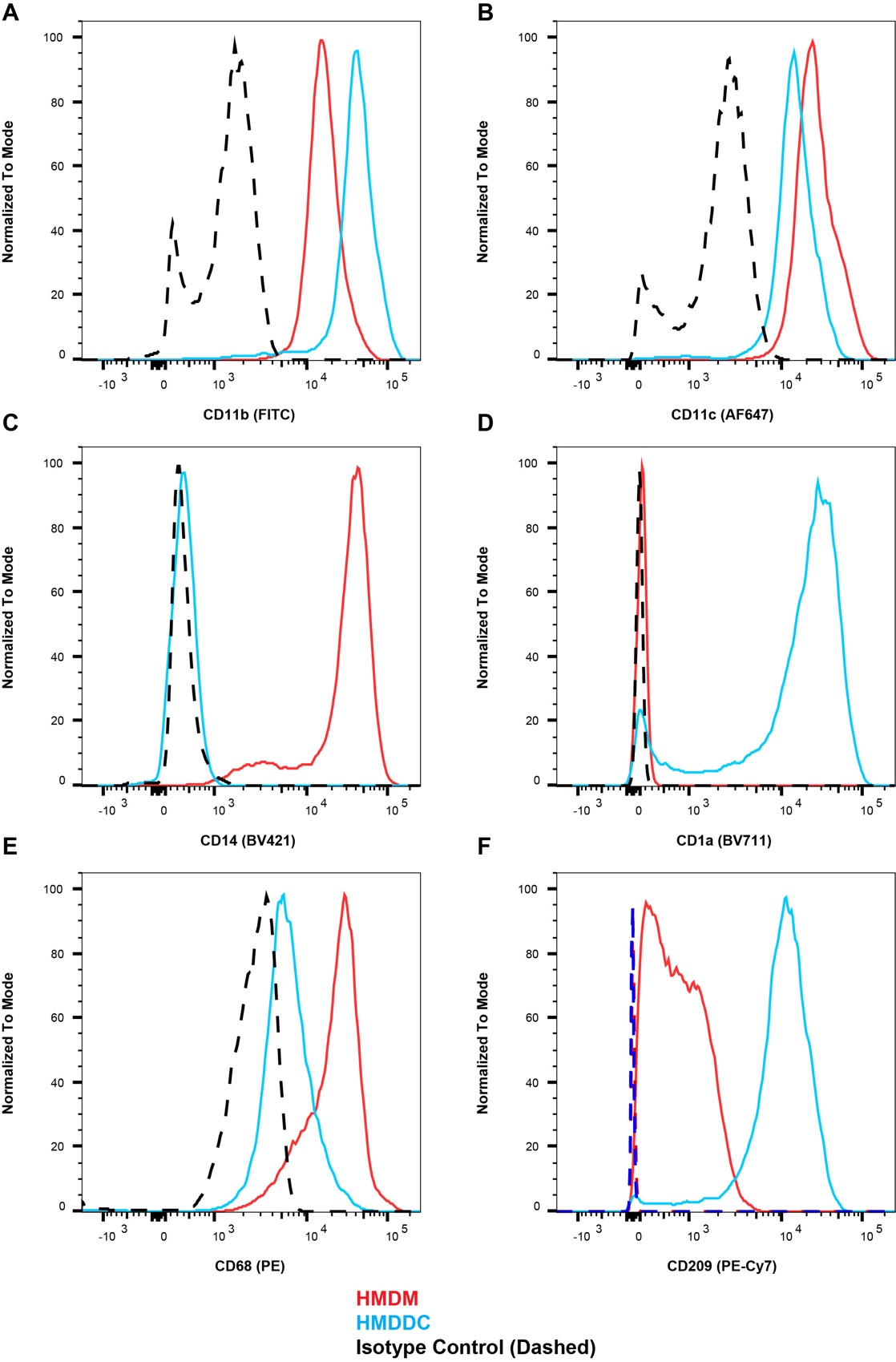


**Supplementary Figure 1.** Primary human monocytes isolated from peripheral blood mononuclear cells can be differentiated into macrophages (HMDMs) and dendritic cells (HMDDCs), and these myeloid cells can be distinguished based on their CD14 and CD1a surface expression. (A) HMDDCs express more CD11b on their surface than HMDMs, as expected. (B) HMDMs express more CD11c on their surface than HMDDCs, as expected. (C) HMDMs express CD14 on their surface while HMDDCs do not, meaning CD14 surface expression can be used to identify macrophages. (D) HMDDCs have high CD1a surface expression while HMDMs do not, meaning CD1a surface expression can be used to identify dendritic cells. (E) HMDMs express more CD68 than HMDDCs, as expected. (F) HMDDCs express more CD209 on their surface than HMDMs, as expected. It should be noted that the dashed line in the CD209 histogram represents the signal from the unstained control, due to the lack of availability of an isotype control during data acquisition. Histograms are representative of data from n = 2 independent donors.


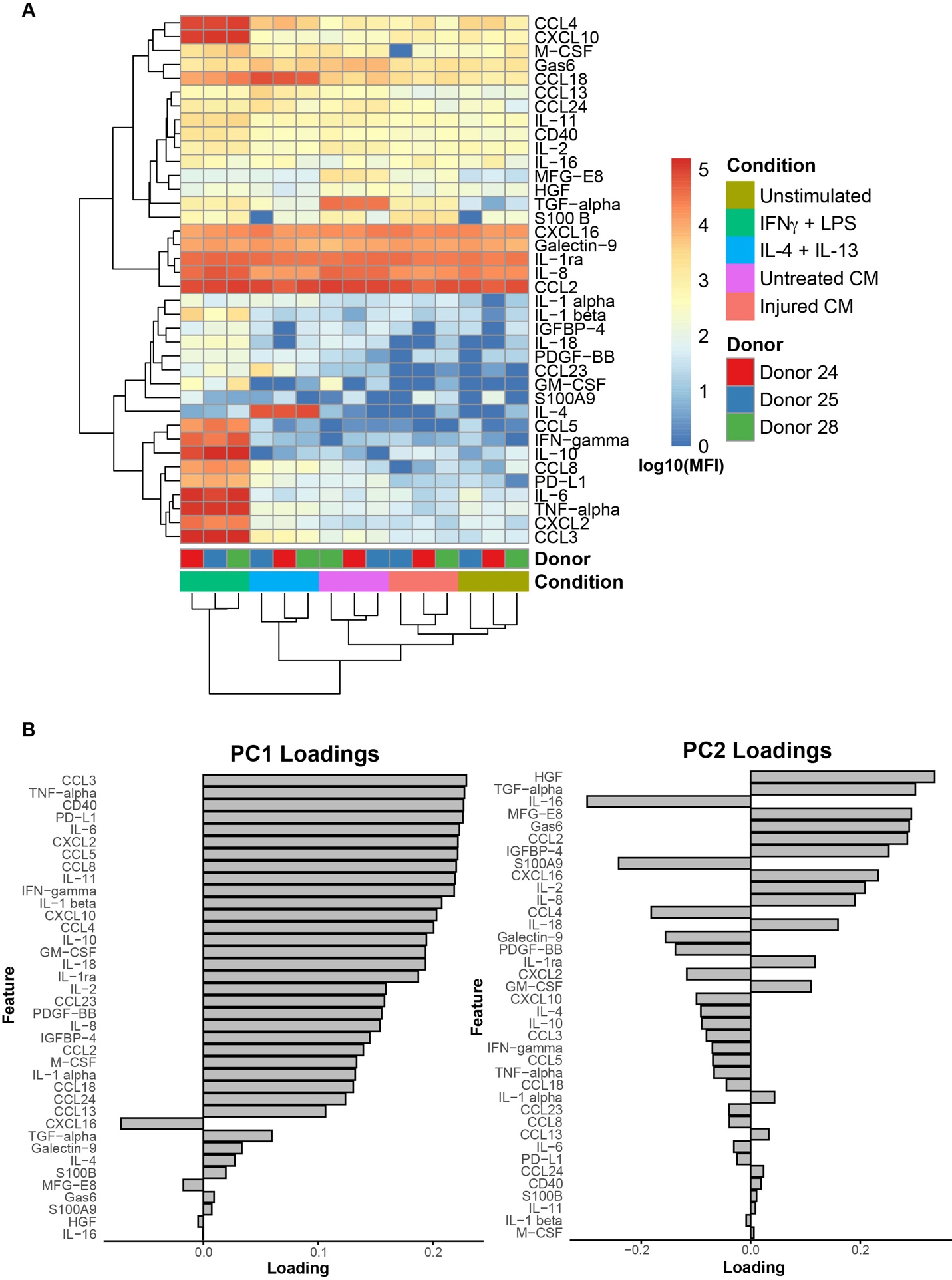


**Supplementary Figure 2.** Cytokines, chemokines, and growth factors detected in HMDM supernatants via Luminex. (A) log_10_(MFI) values that were the input for the PCA shown in Figure 2. (B) Extended PC1 (left) and PC2 (right) loadings plots from Figure 2 with all the features included. n = 3 independent donors, same donors as Figure 2.


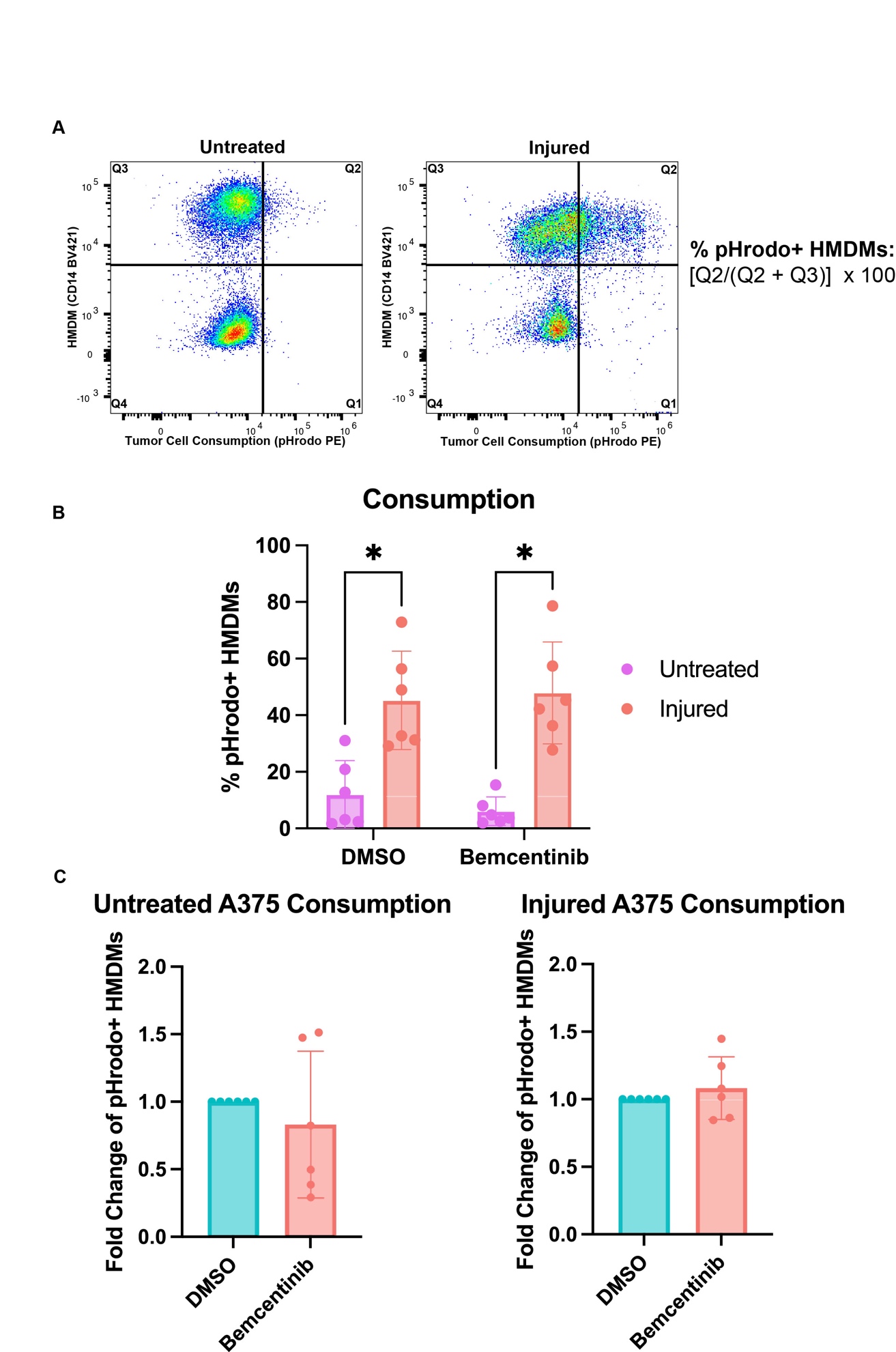


**Supplementary Figure 3.** HMDM consumption of A375s is dictated by A375 state and is not affected by bemcentinib treatment. (A) Representative flow cytometry dot plots and gating of HMDM consumption of untreated (left) and injured (right) A375s. HMDMs can be distinguished from A375s by CD14 surface expression. HMDM consumption of A375s is defined as the percent of pHrodo+ cells out of all of the HMDMs. (B) HMDMs consume more injured A375s than untreated A375s. Mann-Whitney U test with Bonferroni-Dunn correction: *padj < 0.05. (C) Bemcentinib treatment does not affect HMDM consumption of untreated (left) or injured (right) A375s. n = 6 independent donors, same donors as Figure 3.


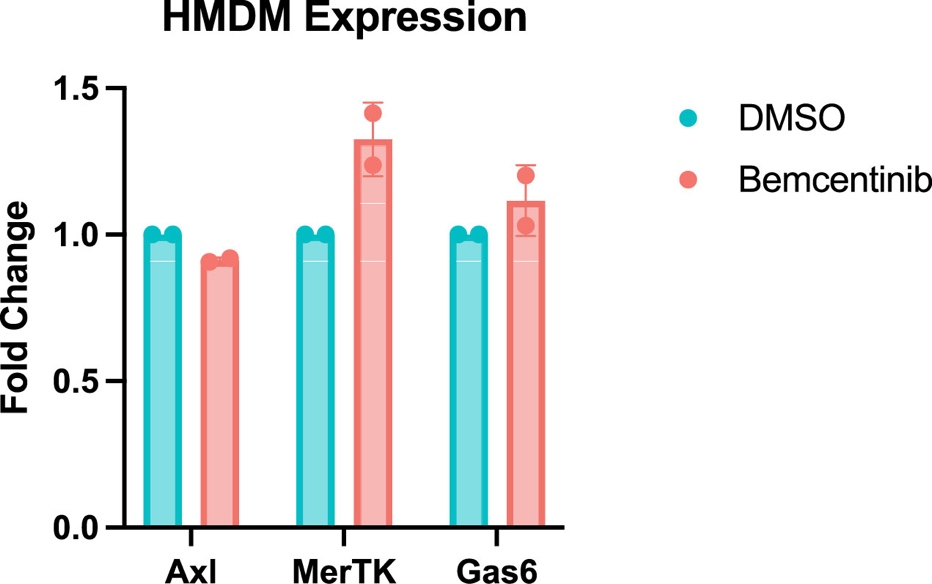


**Supplementary Figure 4.** Bemcentinib treatment may result in increased HMDM expression of MerTK to compensate Axl inhibition, measured via ELISA on cell lysates. n = 2 independent donors.


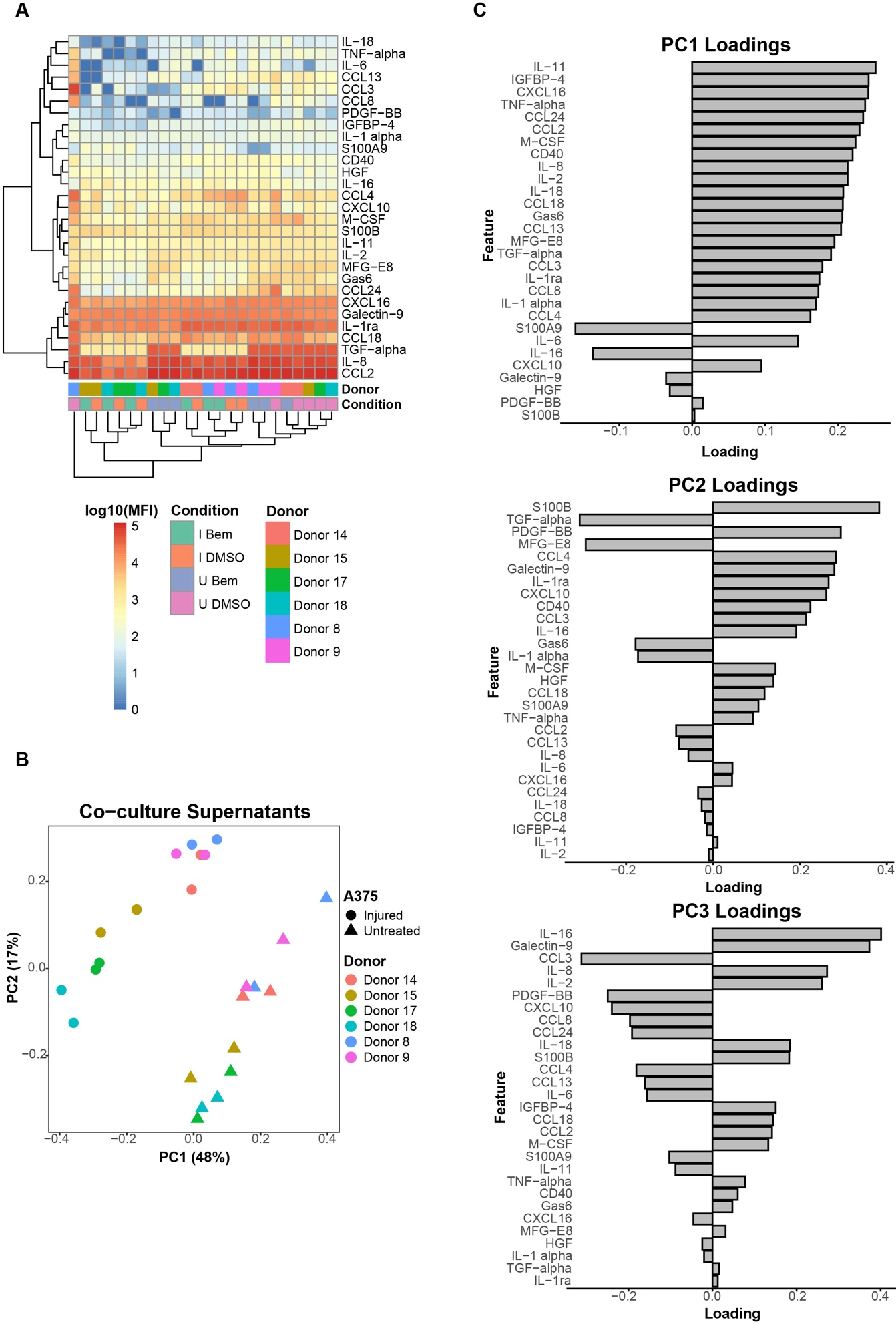


**Supplementary Figure 5.** Cytokines, chemokines, and growth factors detected in co-culture supernatants via Luminex. (A) log_10_(MFI) values that were the input for the PCA shown in Figure 3. (B) PCA scores plot of PC2 against PC1 colored by donor shows donor differences dominate variance captured by PC2. (C) Extended PC1 (top), PC2 (middle), and PC3 (bottom) loadings plots from Figure 3 with all the features included. n = 6 independent donors, same donors as Figure 3. I = injured A375, U = untreated A375, Bem = bemcentinib.


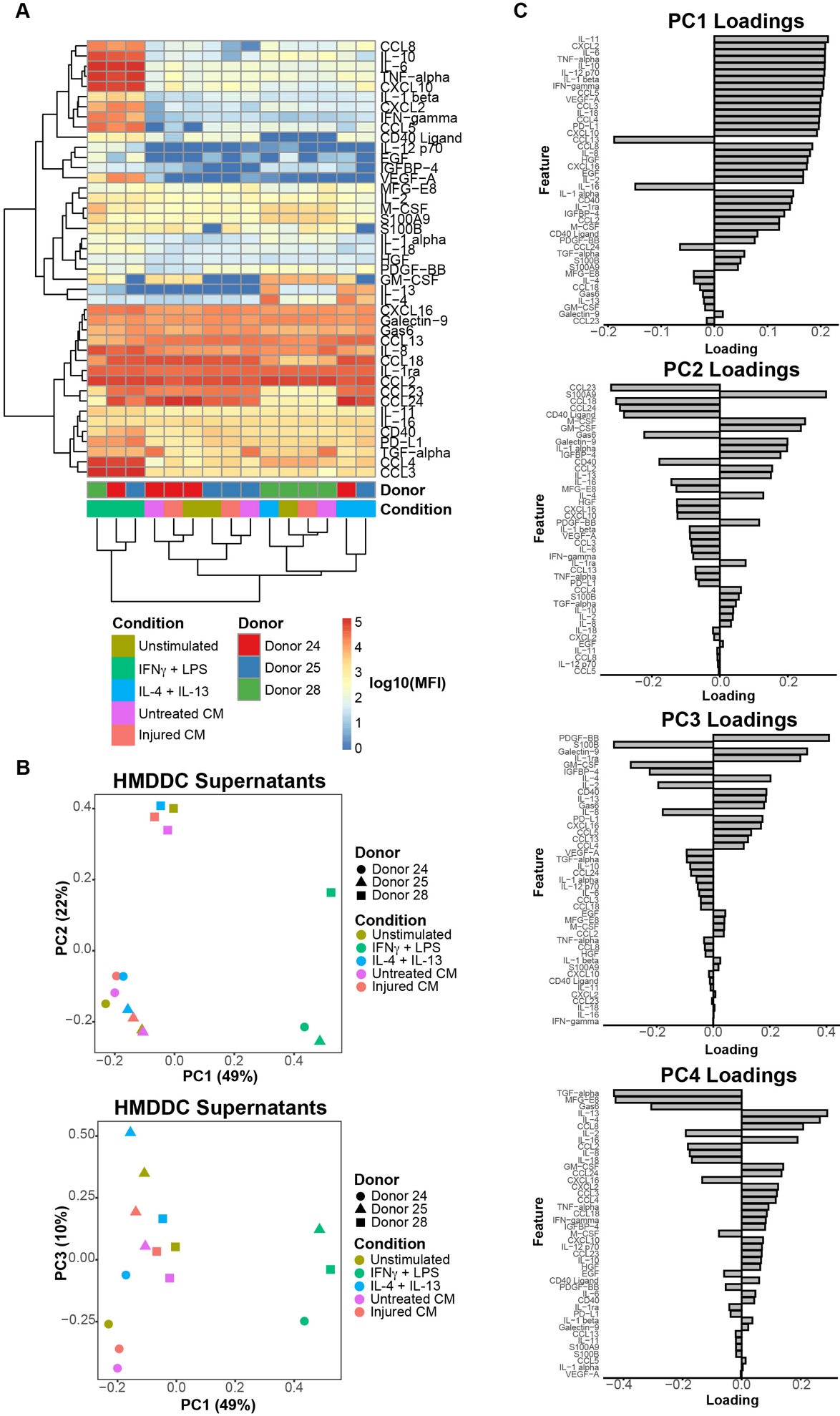


**Supplementary Figure 6.** Cytokines, chemokines, and growth factors detected in HMDDC supernatants via Luminex. (A) log_10_(MFI) values that were the input for the PCA shown in Figure 4. (B) PCA scores plots of PC2 against PC1 (top) and PC3 against PC1 (bottom) show donor differences dominate variance captured by PC2 and PC3. (C) Extended PC1 (top) and PC4 (bottom) loadings plots from Figure 4 with all the features included. PC2 and PC3 loadings plots (middle plots) show which features drive separation along these principal components in (B). n = 3 independent donors, same donors as Figure 2 and Figure 4.


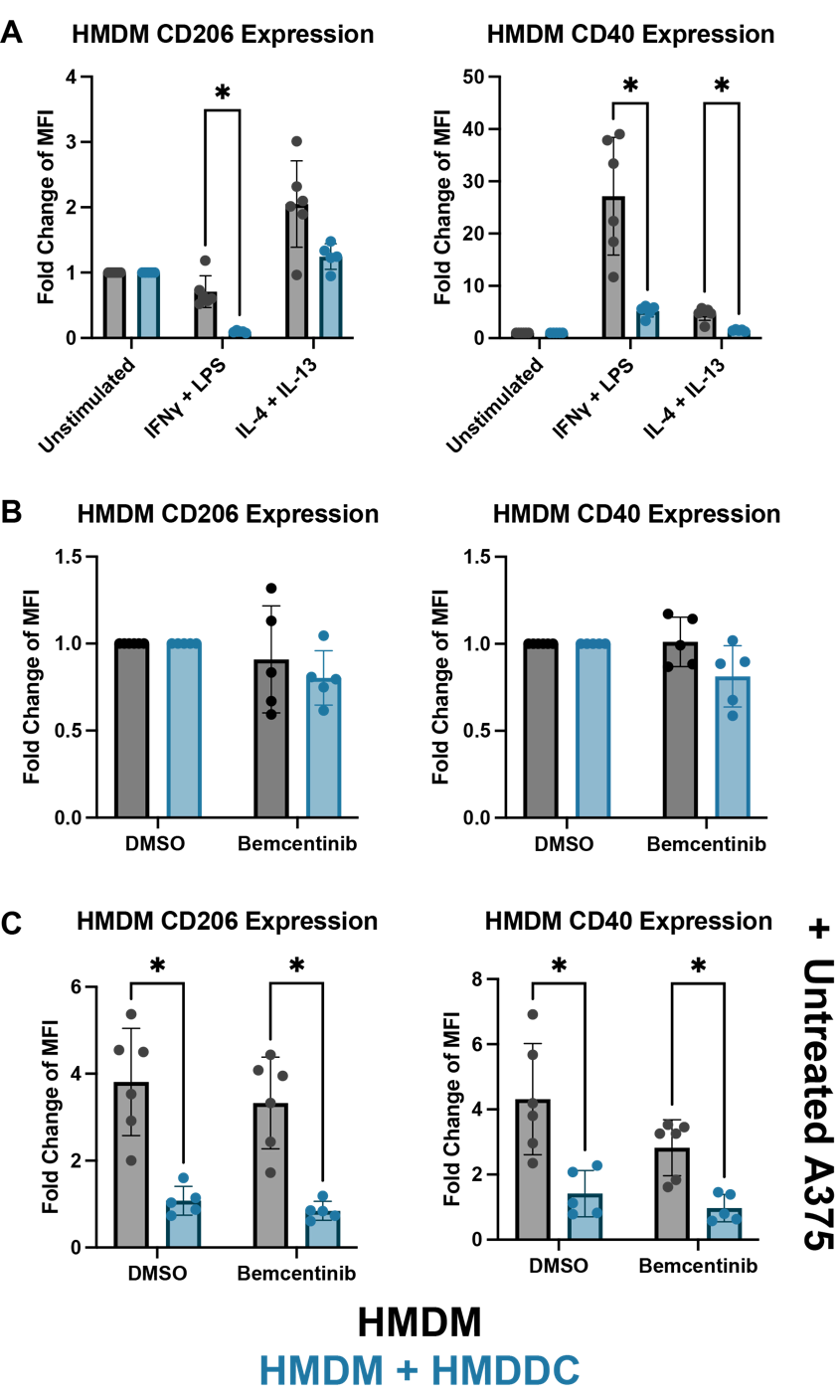


**Supplementary Figure 7.** HMDDCs modulate HMDM response. (A) In the absence of A375s, the addition of dendritic cells decreases macrophage surface expression of CD206 in the IFNγ + LPS condition as well as expression of CD40 in the IFNγ + LPS and IL-4 + IL-13 conditions. (B) In the absence of A375s, the addition of dendritic cells does not impact macrophage surface expression of CD206 or CD40, regardless of treatment with bemcentinib or DMSO vehicle. (C) Regardless of treatment with bemcentinib or DMSO vehicle, the addition of dendritic cells decreases macrophage surface expression of both CD206 and CD40 in the presence of untreated A375s. Mann-Whitney U test with Bonferroni-Dunn correction: *padj < 0.05. n = 5-6 independent donors, same donors as Figure 3, for samples without dendritic cells. n = 5 independent donors, same donors as Figure 5, for samples with dendritic cells. MFI = raw median fluorescent intensity (not batch-corrected).


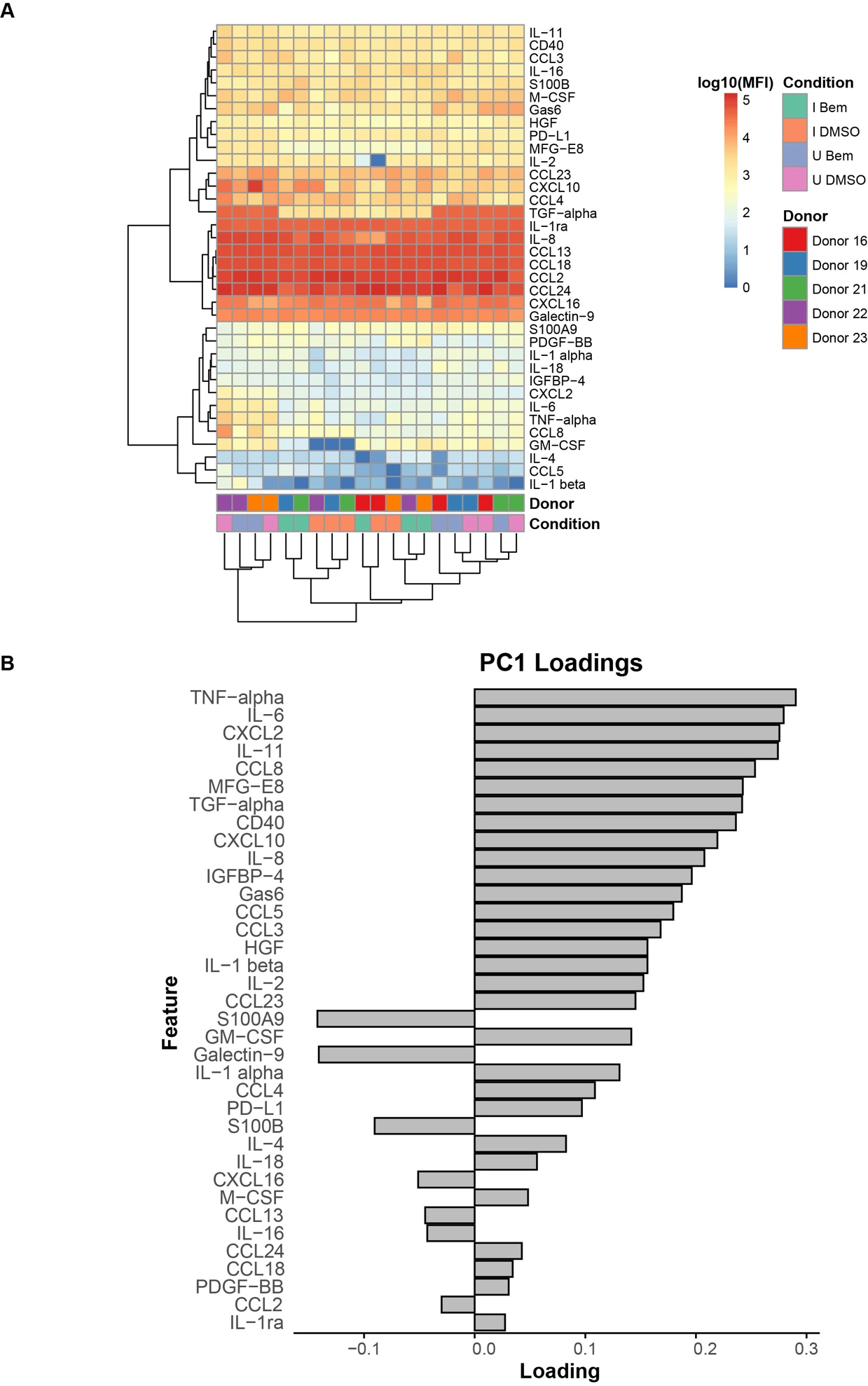


**Supplementary Figure 8.** Cytokines, chemokines, and growth factors detected in tri-culture supernatants via Luminex. (A) log_10_(MFI) values that were the input for the PCA shown in Figure 5. (B) Extended PC1 loadings plot from Figure 5 with all the features included. n = 5 independent donors, same donors as Figure 5. I = injured A375, U = untreated A375, Bem = bemcentinib.


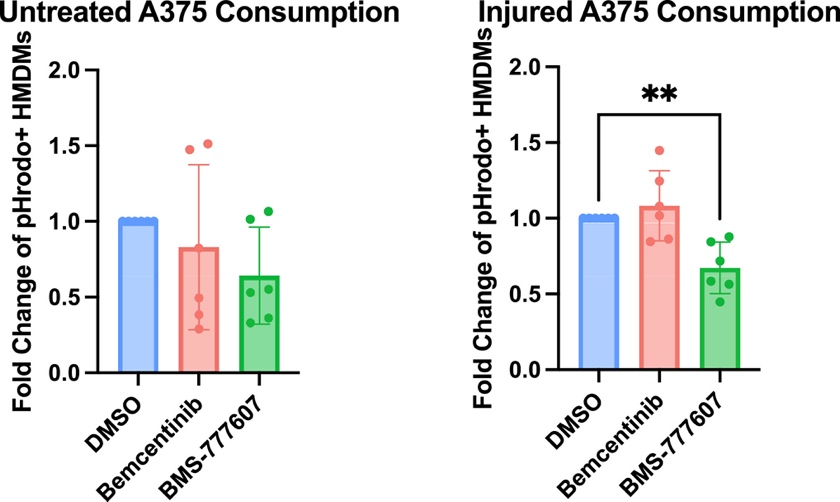


**Supplementary Figure 9.** HMDM consumption of A375s. There is no change in HMDM consumption of untreated A375s with bemcentinib or BMS-777607 treatment (left). BMS-777607 treatment results in decreased HMDM consumption of injured A375s (right). n = 6 independent donors, same donors as Figure 3. One-way ANOVA with post-hoc Dunnett’s test: **p < 0.01.
